# The MdWRKY31 transcription factor binds to the *MdRAV1* promoter to mediate ABA sensitivity

**DOI:** 10.1038/s41438-019-0147-1

**Published:** 2019-06-01

**Authors:** Xian-Yan Zhao, Chen-Hui Qi, Han Jiang, Chun-Xiang You, Qing-Mei Guan, Feng-Wang Ma, Yuan-Yuan Li, Yu-Jin Hao

**Affiliations:** 1 State Key Laboratory of Crop Stress Biology for Arid Areas/Shaanxi Key Laboratory of Apple, College of Horticulture, Northwest A&F University, Yangling, 712100 Shaanxi China; 2 National Key Laboratory of Crop Biology, College of Horticulture Science and Engineering, Shandong Agricultural University, Tai-An, Shandong 271018 China

**Keywords:** Transcriptional regulatory elements, Plant signalling, Plant signalling, Transcriptional regulatory elements, Transcriptional regulatory elements

## Abstract

The phytohormone abscisic acid (ABA) is a major element involved in apple (*Malus domestica*) production because of its role in seed germination and early seedling development. The WRKY family, which is one of the largest families of transcription factors, plays an important role in ABA signaling in plants. However, the underlying molecular mechanisms of WRKY-mediated ABA sensitivity in apple are poorly understood. A genome-wide transcriptome analysis indicated that *MdWRKY31* is a key factor induced by ABA. Quantitative real-time PCR showed that *MdWRKY31* is induced by ABA in response to PEG4000, which is used to simulate drought. As a transcription factor, MdWRKY31 is localized in the nucleus. Ectopic expression of *MdWRKY31* in *Arabidopsis* and *Nicotiana benthamiana* enhanced plant sensitivity to ABA. Overexpression of *MdWRKY31* in apple roots and apple calli increased sensitivity to ABA, whereas repression of *MdWRKY31* reduced sensitivity to ABA in the roots of ‘Royal Gala’. Electrophoretic mobility shift assays, chromatin immunoprecipitation PCR, and yeast one-hybrid assays indicated that MdWRKY31 directly binds to the promoter of *MdRAV1*. Expression analyses of transgenic apple calli containing *MdWRKY31* and *pMdRAV1::GUS* implied that MdWRKY31 represses the expression of *MdRAV1*. We also found that MdRAV1 binds directly to the promoters of *MdABI3* and *MdABI4* and repressed their expression. Our findings reveal a new important regulatory mechanism of MdWRKY31-MdRAV1-MdABIs in the ABA signaling pathway in apple.

## Introduction

Apple fruit quality and yield are affected by cultivars and various environmental factors. Among the environmental factors, phytohormones greatly influence plant development and productivity and play vital roles in the chlorophyll content, alterations to nutrients and moisture, the accumulation of harmful substances, and insect pests. As central integrators, plant hormones are involved in complex developmental and stress-adaptive signaling cascades throughout plant responses involving signaling of the phytohormone abscisic acid (ABA). ABA plays a vital role in the activation of plant cellular responses to stress and is a central regulator of growth inhibition^1–3^. ABA also influences plant growth and development. At the ripening stage of ‘Orin’ apples, ABA promotes the biosynthesis of aromatic esters related to ethylene^4^. Studies have also shown that ABA plays an important role in sugar accumulation in fleshy fruits^5^.

ABA is involved in plant growth via a highly complex network; ABA is mainly a signaling pathway component. In the past few decades, the mechanism of the ABA signaling has been clearly revealed. ABA receptors, which perceive the initial activity, sense the ABA signal and elicit downstream signaling cascades to evoke physiological responses. ABA receptors include plasma membrane and intracellular receptors^6–8^. An unconventional G protein-coupled receptor (GCR2) and a novel class of G protein-coupled receptors (GTG1 and GTG2), which are plasma membrane ABA receptors, have been identified^9–11^. PYR/PYL/RCAR proteins, which are considered cytosolic ABA receptors, mediate downstream gene expression of the ABA signaling pathway by directly inhibiting type 2C protein phosphatases^12–14^. Because of the complex network of ABA signaling, the functions of additional proteins in the ABA signaling pathway remain unclear.

As one of the largest transcription factor families, the WRKY family of proteins is associated with many stress response defense pathways because of the highly conserved WRKY domain of these proteins that binds the W-box (T)(T)TGAC(C/T)^15,16^. WRKY transcription factors are involved in the ABA signaling pathway. AtWRKY40 is related to the ABA signal by negatively regulating the expression of *ABI4* and *ABI5*, which encode APETALA2 domain-containing and basic leucine zipper (bZIP)-type transcription factors, respectively^17,18^. During seed germination and early seedling development, *abi4* and *abi5* mutants exhibit ABA-insensitive phenotypes^17^. AtWRKY18 and AtWRKY60, which belong to the same group as AtWRKY40, play negative roles in ABA signaling^19^. During seed germination and vegetative growth, *AtWRKY63* and *wrky2* mutants exhibit similar hypersensitive symptoms in response to exogenous ABA^20,21^. In seed dormancy, AtWRKY41 participates in ABA signaling by regulating the direct expression of *ABI3*^22^. However, AtWRKY8 protects plants against TMV-cg by affecting ethylene and ABA signaling^23^.

Recently, AtWRKY6 was found to be a positive regulator of ABA signaling by directly regulating *RAV1* expression during seed germination and early seedling development^24^. In *Arabidopsis*, AtWRKY6 is induced significantly by ABA^24^, which clusters into the same group as does AtWRKY31^15^. *AtRAV1* overexpression slows rosette leaf and lateral root development, whereas suppressing *AtRAV1* expression results in an early-flowering phenotype^25^. AtRAV1 negatively regulates plant development and positively regulates leaf senescence by causing a premature phenotype^26^. AtRAV1 thus plays a vital role in ABA signaling by repressing the expression of *ABI3*, *ABI4*, and *ABI5*^27^.

Many WRKYs play crucial roles in response to ABA^28^; therefore, it is worthwhile to elucidate the functions and regulatory mechanisms of MdWRKYs in the ABA signaling pathway in apple. In this study, we found MdWRKY31 to be a positive regulator of ABA signaling. Ectopic expression of *MdWRKY31* in *Arabidopsis* and *Nicotiana benthamiana* exhibited ABA-hypersensitive phenotypes during seed germination and early seedling development. When *MdWRKY31* was overexpressed in apple seedlings and calli, the transgenic lines showed hypersensitive symptoms similar to those in response to ABA by elevating ABA-responsive genes. Correspondingly, repressing the expression of *MdWRKY31* reduced sensitivity to ABA. By binding directly to its promoter, MdWRKY31 was able to repress the expression of *MdRAV1*. In addition, MdRAV1 could bind to the promoters of *MdABI3* and *MdABI4* to inhibit their expression directly. Our findings reveal the function and a novel molecular mechanism of MdWRKY31 in mediating the ABA signaling pathway in apple.

## Results

### Expression analysis of *MdWRKY31*

**Fig. 1:**
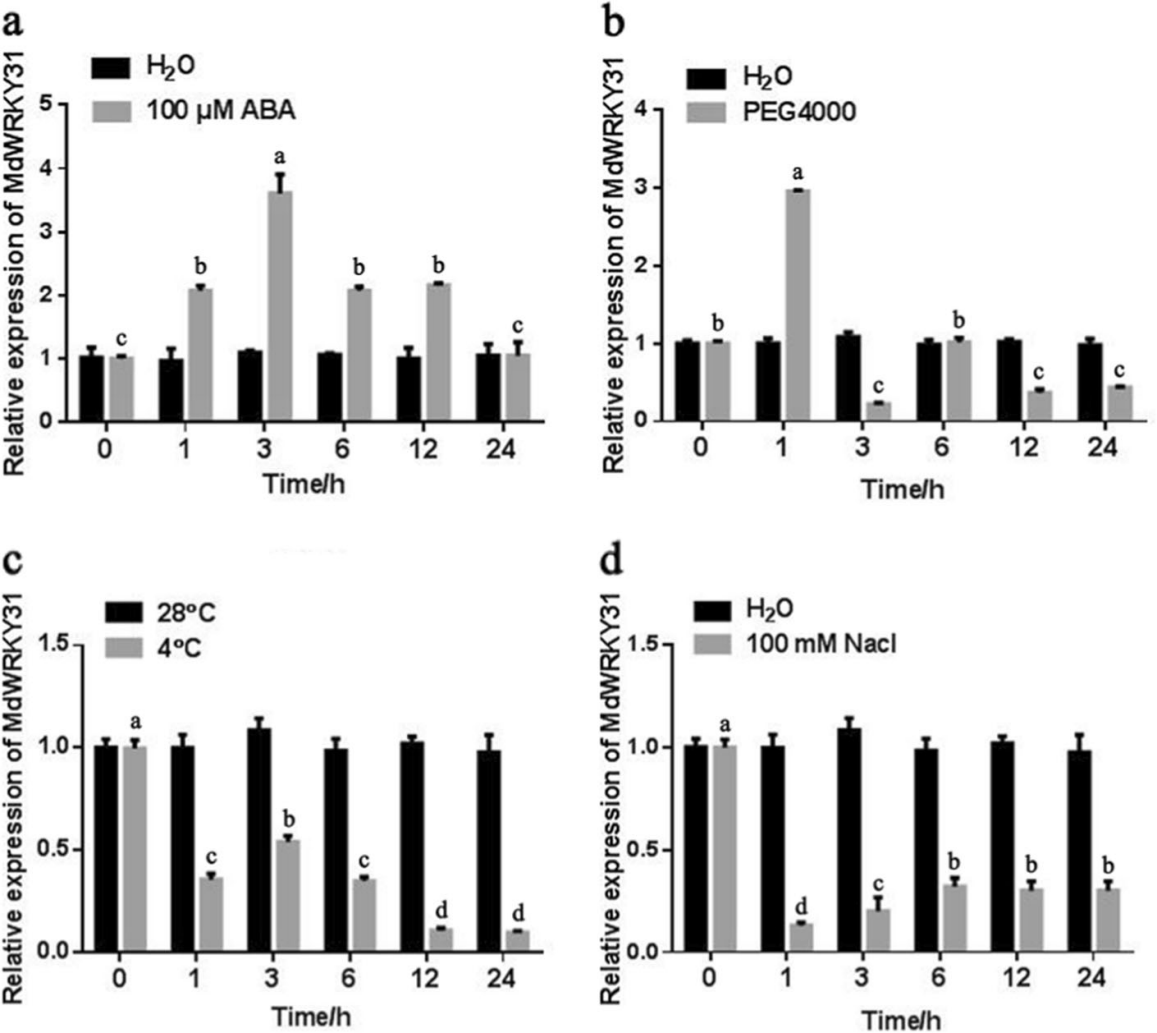
Expression pattern of *MdWRKY31* in response to abiotic stresses. **a**–**d** *MdWRKY31* transcript levels in tissue cultures of ‘Royal Gala’ treated with 100 μM ABA, PEG4000, 4 °C, and 100 mM NaCl, respectively. H_2_O (**a**, **b**, **d**) treatment and 28 °C (**c**) were used as controls; MdACTIN was an internal reference. The data were replicated three times, and the bars indicate SEs. Mean differences are significant at the *P*_0.05_ level between the bars with different letters and not significant at the *P*_0.05_ level with the same letters. The same setup applies to the following figure

ABA is an important phytohormone that plays a crucial role in plant growth and development. To identify potential genes associated with ABA, we performed an RNA-seq analysis to examine the differentially expressed genes in ‘Royal Gala’ seedlings treated with 100 μM ABA or in seedlings under normal conditions. The data revealed that many genes were either upregulated or downregulated (Table S2). Among them, an apple WRKY transcription factor (MD05G1349800) was identified to be apparently induced by ABA treatment; this transcription factors was labeled MdWRKY31. To further verify the RNA-seq results, the expression of *MdWRKY31* in ‘Royal Gala’ seedlings treated with 100 μM ABA for 0, 1, 3, 6, 12, and 24 h was measured. The results indicated that the transcription of *MdWRKY31* was induced by ABA, peaking after 3 h of treatment and then declining gradually (Fig. 1a). We also examined the transcript level of *MdWRKY31* under abiotic stress, including polyethylene glycol (PEG), low temperature, and salt (NaCl) stress, and found that PEG4000 treatment also induced the expression of *MdWRKY31* (Fig. 1b). However, *MdWRKY31* expression was strongly suppressed by low temperature and salt treatment (Fig. 1c, d). These results suggest that MdWRKY31 responded to various stresses and might function during these processes.

### Isolation and analysis of MdWRKY31

**Fig. 2:**
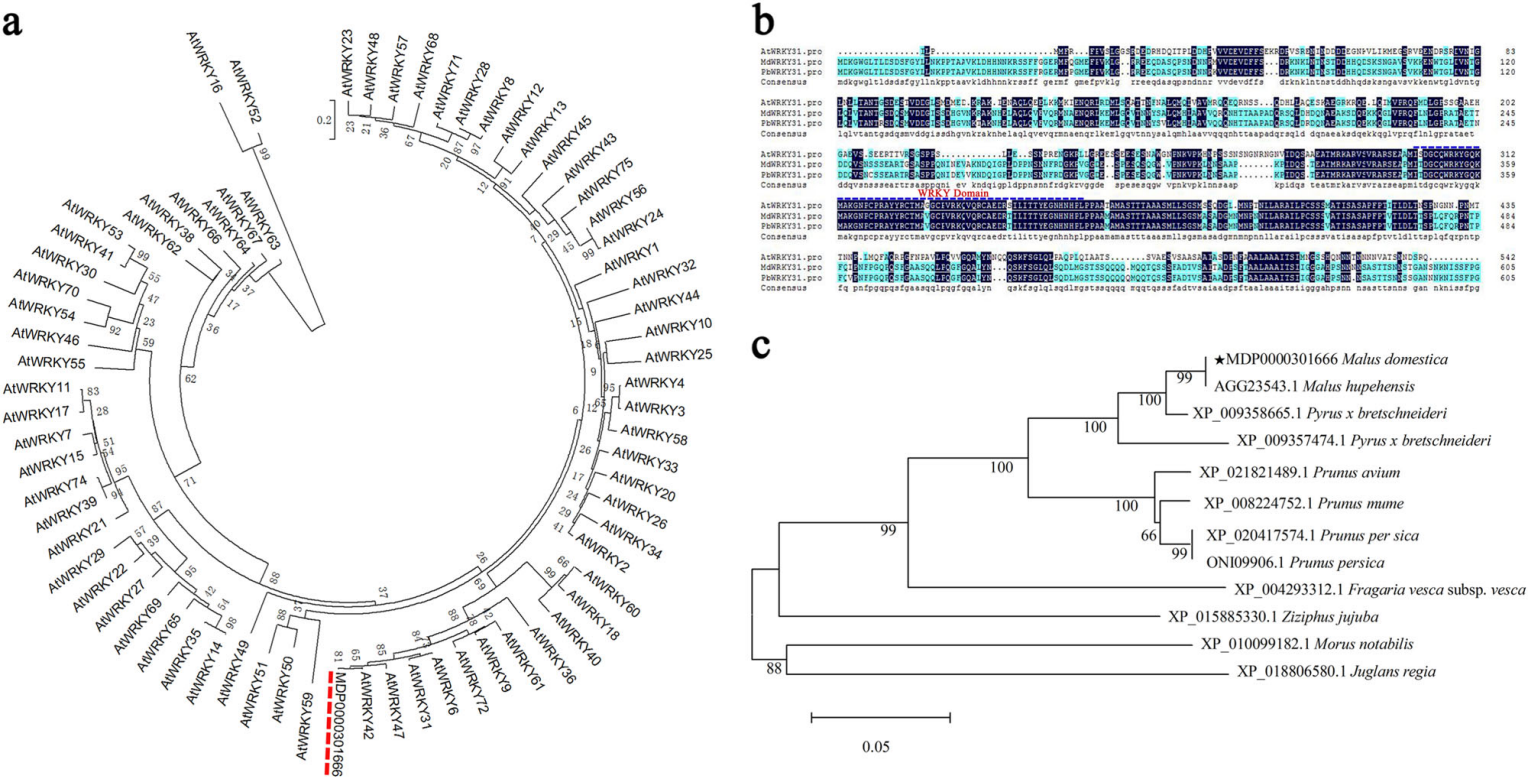
Sequence analysis of the MdWRKY31 protein. **a** Multiple sequence alignment of MdWRKY31 and WRKYs from Arabidopsis. **b** Alignment of three WRKY proteins. WRKY domains are indicated by dotted lines. At: *Arabidopsis thaliana,* Md *Malus domestica,* Pb *Pyrus bretschneideri*. **c** Multiple sequence alignment of MdWRKY31 and WRKYs from other plant species

To verify the function of MdWRKY31, RNA was extracted from tissue-cultured ‘Royal Gala’ seedlings. A cDNA template was obtained from RNA reverse transcription. The full-length sequence of 1821 bp of *MdWRKY31* was amplified with upstream and downstream primers of *MdWRKY31* (MdWRKY31F/R). *MdWRKY31* encodes a protein of 607 amino acid residues with a complete ORF and an isoelectric point of 7.19. Sequence analysis between MdWRKY31 and 71 WRKYs in *Arabidopsis* showed that MdWRKY31 clustered in a group together with AtWRKY42, AtWRKY47, AtWRKY31, and AtWRKY6 (Fig. 2a). Multiple sequence alignment of WRKY31 from *Arabidopsis*, *Pyrus bretschneideri*, and *Malus domestica* indicated that the MdWRKY31 protein contained a conserved WRKY domain in its C-terminal region (Fig. 2b). An evolutionary tree was subsequently constructed to analyze MdWRKY31 and other WRKYs from different plant species, including *Malus hupehensis*, *Pyrus bretschneideri*, *Prunus avium*, *Prunus mume*, *Prunus persica*, *Fragaria vesca* subsp. *Vesca*, *Ziziphus jujube*, *Morus notabilis*, and *Juglans regia*. Two apple WRKY TFs from *Malus domestica* and *Malus hupehensis* demonstrated more than 99% sequence similarity. The apple MdWRKY31 protein exhibited the closest relationship to the pear WRKY31 protein and the farthest relationship with the walnut WRKY31 protein (Fig. 2c).

### MdWRKY31 localizes to the nucleus

**Fig. 3:**
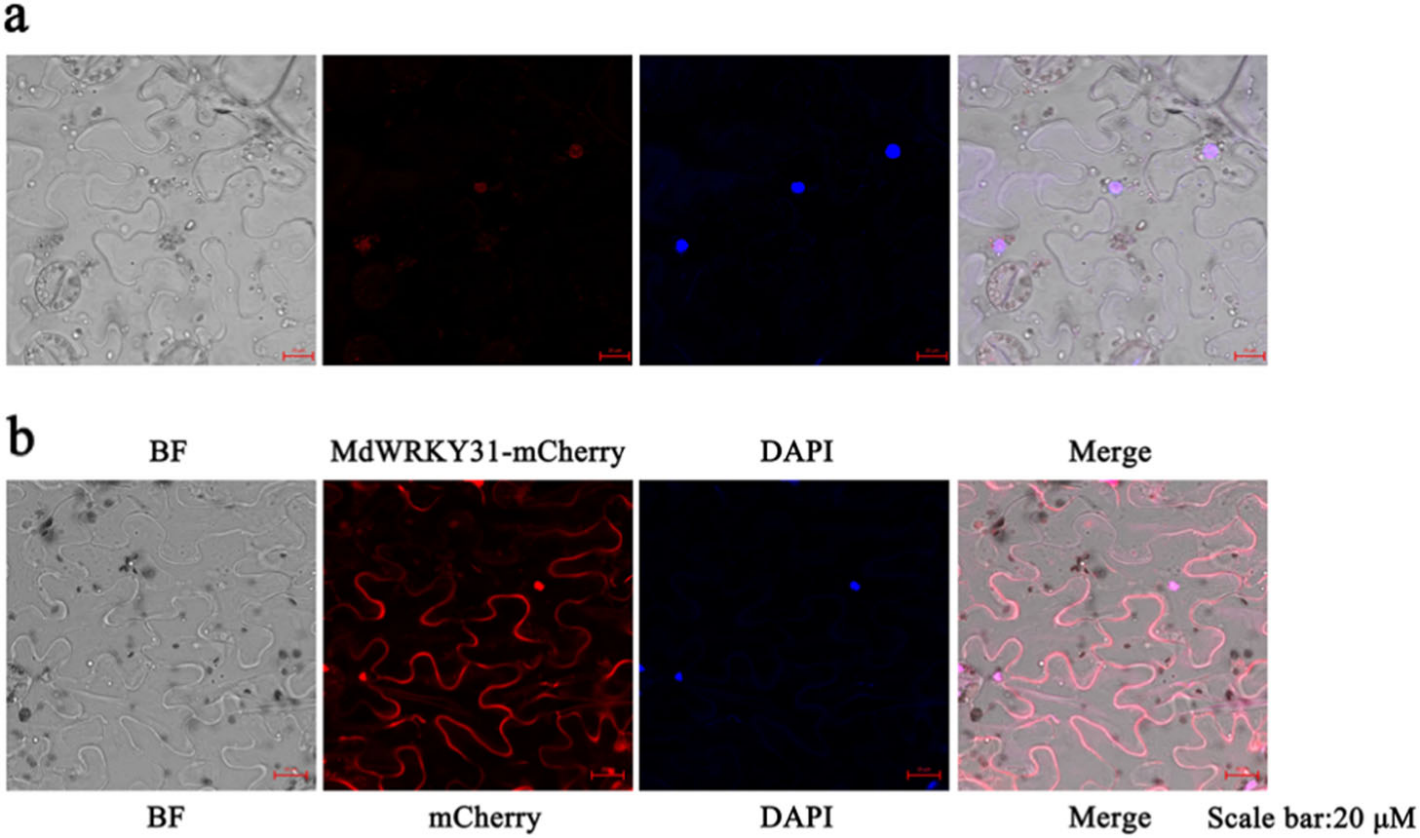
Subcellular localization of the MdWRKY31 protein. Laser-scanning confocal microscopy images of *Nicotiana benthamiana* leaves expressing *MdWRKY31-RFP*. The micrographs show *MdWRKY31-RFP* localized to the nucleus. An empty RFP was used as a control. DAPI is a nuclear dye. The scale bar represents 20 μM

Protein function is closely related to cellular location. To examine the cellular localization of the MdWRKY31 protein, a MdWRKY31-mCherry fusion protein whose C-terminal expressed an enhanced red fluorescent protein (ERFP) was constructed. A plasmid containing RFP alone was used as a control. After transient injection into *Nicotiana benthamiana*, red fluorescence of MdWRKY31-mCherry was observed in the nucleus, whereas the fluorescence of the control RFP was uniformly distributed across a greater field of vision; thus, MdWRKY31 was localized to the nucleus (Fig. 3).

### Ectopic expression of *MdWRKY31* in Arabidopsis and tobacco increases sensitivity to ABA

**Fig. 4:**
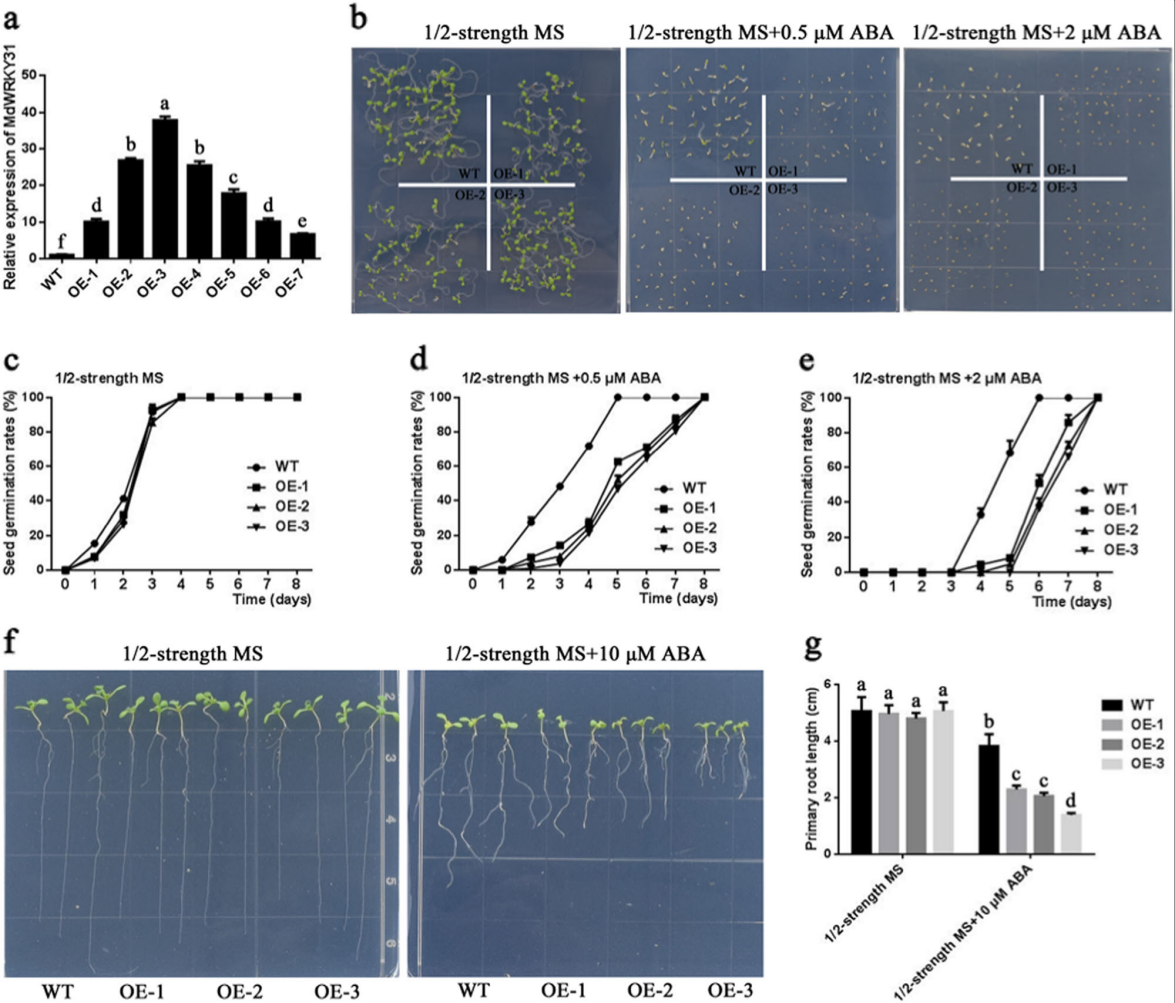
Sensitivity to ABA after *MdWRKY31* ectopic expression in *Arabidopsis*. **a** Determination of the *MdWRKY31* protein in transgenic Arabidopsis. WT represents wild-type *Arabidopsis* (Columbia), and *OE* represents *MdWRKY31*-overexpressing Arabidopsis. The numbers 1–7 represent different transgenic lines. **b** Phenotypes of different types of *Arabidopsis* treated with or without ABA in 1/2-strength MS medium. **c**–**e** Seed germination rates in different types of *Arabidopsis* growing in 1/2-strength MS medium with or without ABA. **f** Phenotypes of different types of *Arabidopsis* growing in 1/2-strength MS medium with or without 10 μM ABA. **g** Length of primary roots treated with or without 10 μM ABA

**Fig. 5:**
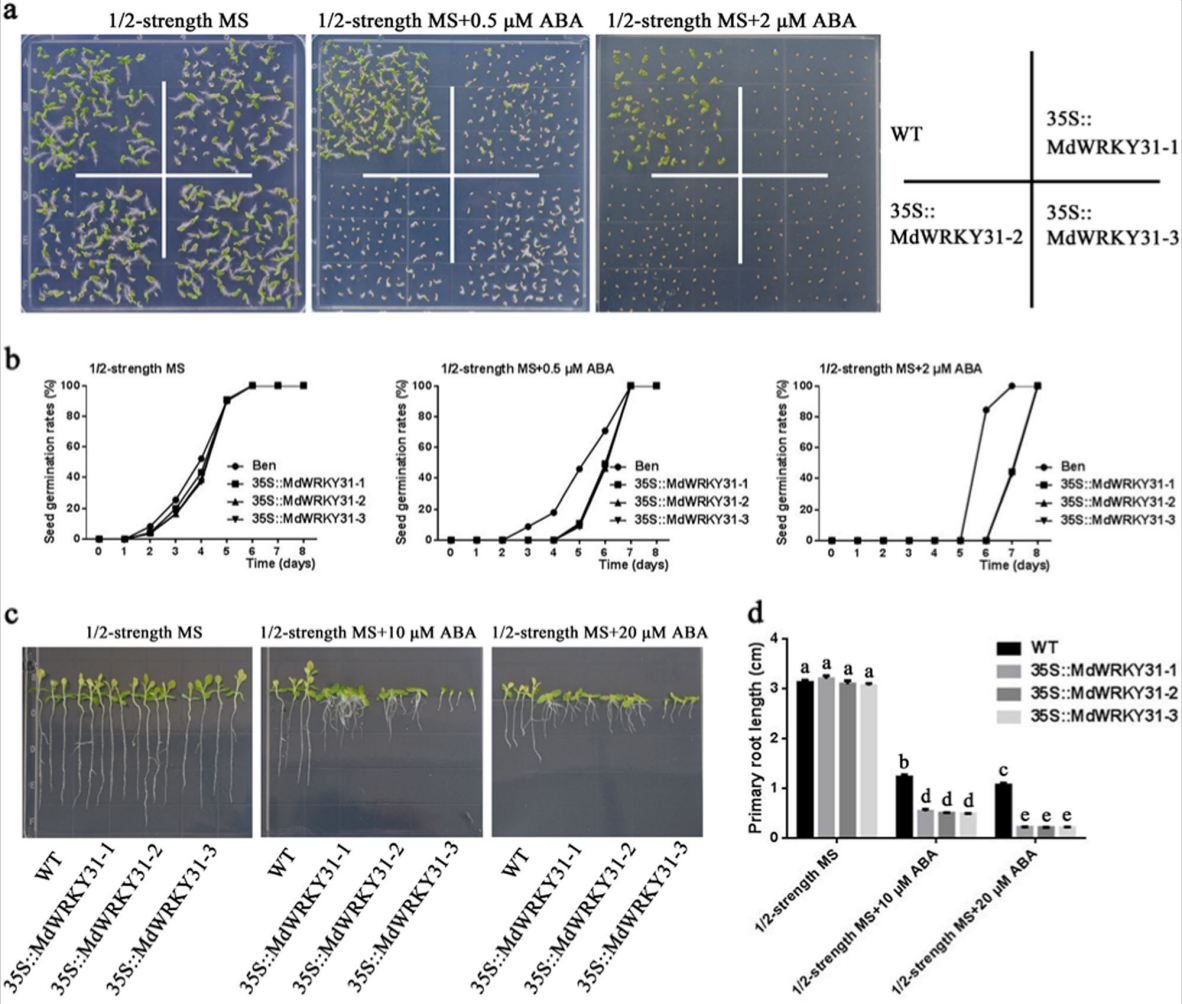
*MdWRKY31* overexpression conferred increased ABA sensitivity to *Nicotiana benthamiana*. **a** Phenotypes of different types of *Nicotiana benthamiana* treated with or without ABA in 1/2-strength MS medium. WT represents wild-type tobacco (Ben). *35::MdWRKY31* represents an MdWRKY31-overexpressing tobacco. The numbers 1–3 represent different transgenic lines. **b** Seed germination rates in different types of *Nicotiana benthamiana* growing in 1/2-strength MS medium with or without ABA. **c** Phenotypes of different types of *Nicotiana benthamiana* growing in 1/2-strength MS medium with or without 10 μM ABA. **d** Length of primary roots treated with or without 10 μM ABA

To determine the function of MdWRKY31, transgenic *Arabidopsis* and *Nicotiana benthamiana* plants overexpressing *MdWRKY31* were obtained (Fig. 4a; Fig. S1). Seven transgenic *Arabidopsis* plants and five transgenic tobacco plants overexpressing *MdWRKY31* presented different *MdWRKY31* expression levels. Three transgenic lines (OE-1, OE-2, and OE-3 in *Arabidopsis*; 35::MdWRKY31-1, 35::MdWRKY31-2 and 35::MdWRKY31-3, respectively) with different *MdWRKY31* gene expression levels (low, moderate, and high, respectively) were selected to perform subsequent phenotypic experiments involving ABA responses. Both the Arabidopsis and tobacco transgenic lines had a lower seed germination rate than did the wild type in response to ABA (Figs. 4b–e and 5a, b). With higher ABA concentrations, seeds of Arabidopsis and tobacco germinated relatively later. Root growth in response to ABA was subsequently examined in WT and MdWRKY31-overexpressing Arabidopsis and tobacco. We found the primary root length to be much shorter in the transgenic lines compared to the wild-type lines in both Arabidopsis and tobacco (Figs. 4f, g and 5c, d). These findings suggested that *MdWRKY31* increased sensitivity to ABA in *Arabidopsis* and *Nicotiana benthamiana*.

### MdWRKY31 positively regulates ABA sensitivity in apple

**Fig. 6:**
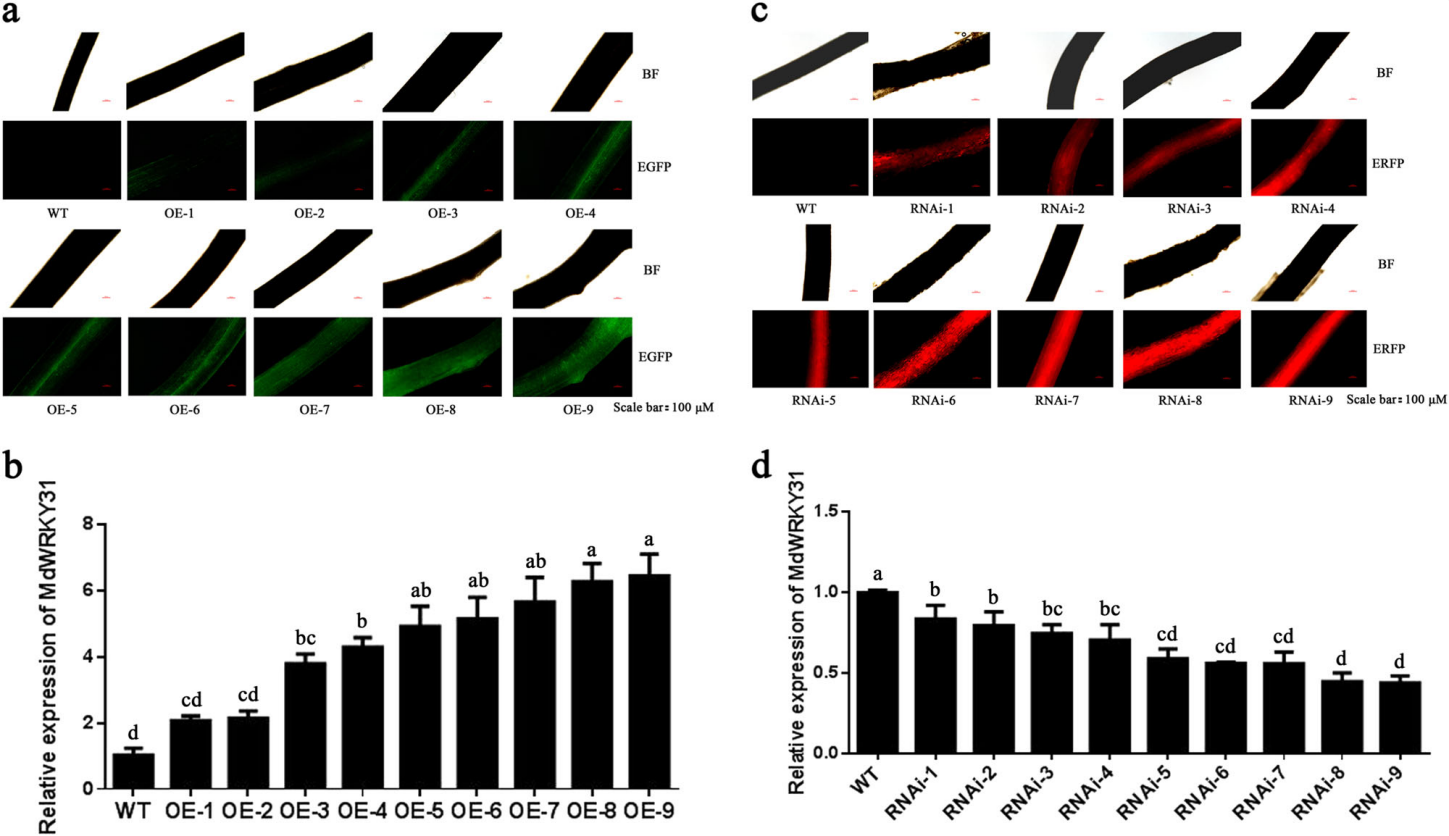
MdWRKY31 overexpression and repression in apple roots. **a** Relative fluorescence intensity of green fluorescent protein (GFP) in the roots. WT represents an empty vector containing a GFP tag; OE represents *MdWRKY31*-overexpressing transgenic apple roots. The numbers 1–9 represent different strains. **b** qRT-PCR determination of *MdWRKY31*-overexpressing roots. **c** Relative fluorescence intensity of red fluorescent protein (RFP) in the roots. WT represents an empty vector containing an RFP tag; RNAi represents *MdWRKY31* repressing roots. The numbers 1–9 represent different strains. **d** qRT-PCR determination of *MdWRKY31*-suppressing roots

**Fig. 7:**
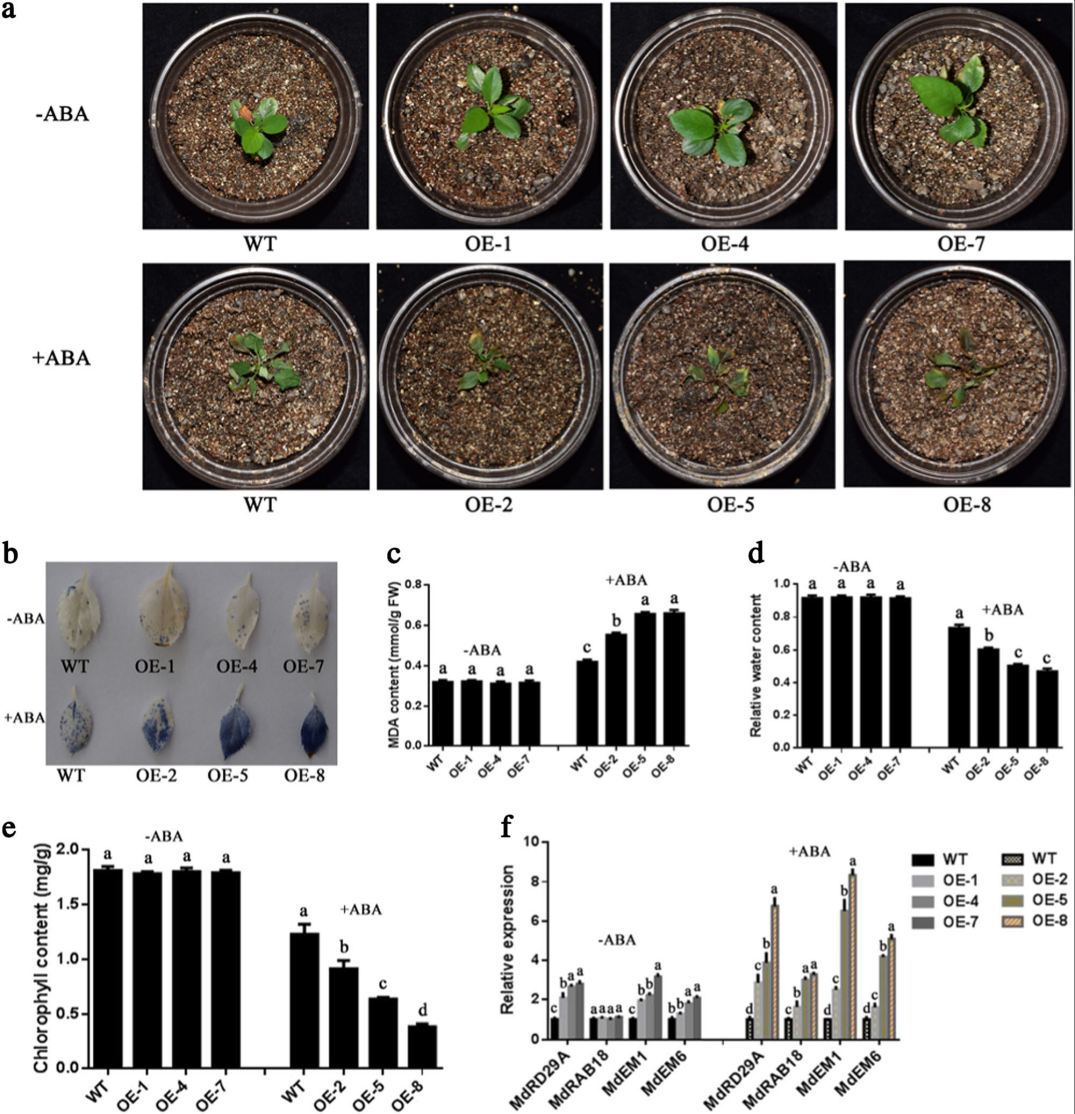
ABA sensitivity of *MdWRKY31*-overexpressing apple roots. **a** Phenotypes of plants transformed with an empty vector that contains a GFP tag (WT) and of six overexpression lines (OE-1, 4, and 7 without ABA treatment and OE-2, 5, and 8 with ABA treatment). **b** NBT staining dye in the leaves of transgenic lines with or without ABA treatment. **c**–**e** MDA content, relative water content, and chlorophyll content of transgenic lines with or without ABA treatment. **f** Expression of four ABA-responsive genes, *MdRD29A*, *MdRAB18*, *MdEM1*, and *MdEM6*, in transgenic lines with or without ABA treatment

**Fig. 8:**
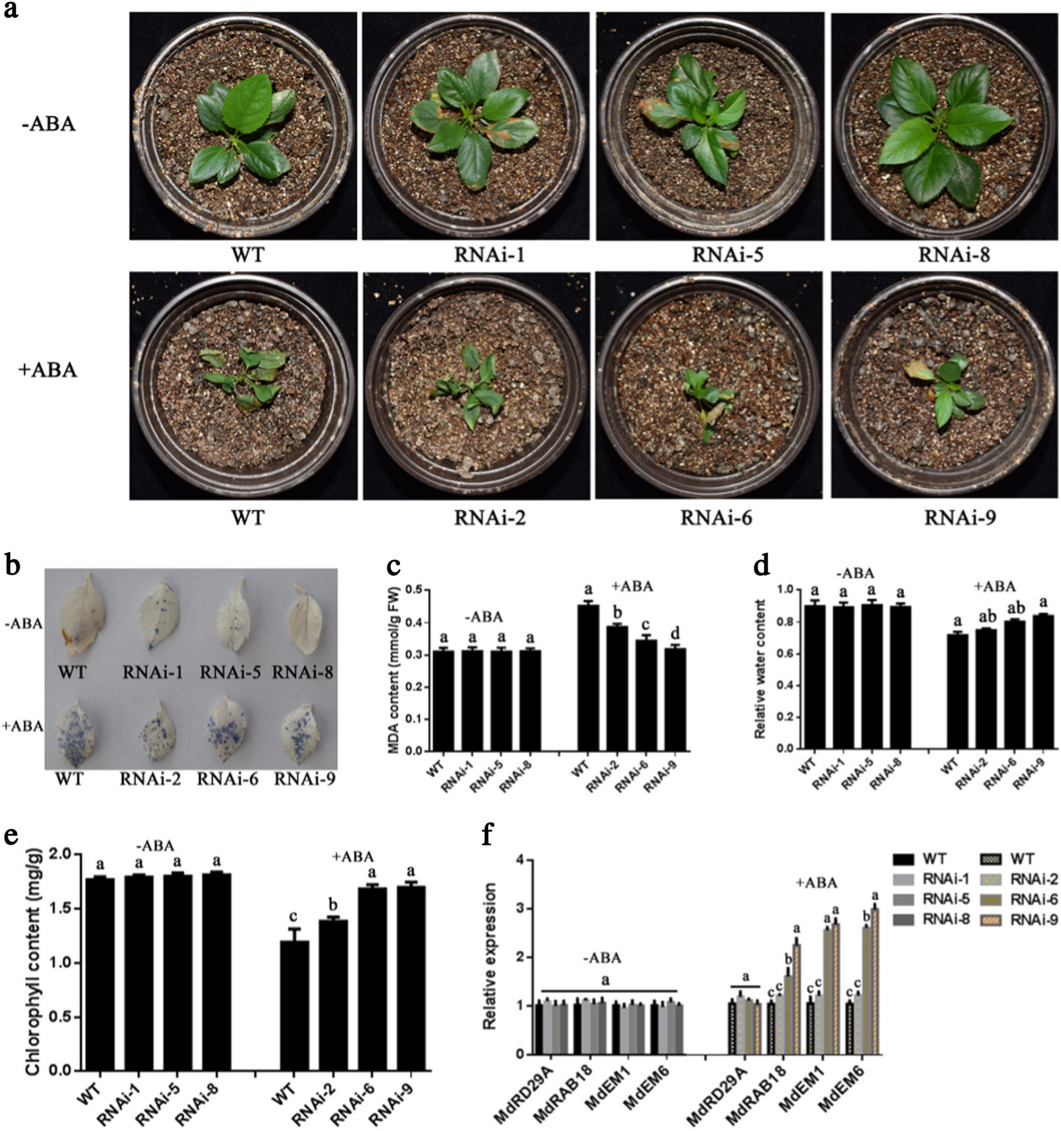
ABA sensitivity of *MdWRKY31*-repressing apple roots. **a** Phenotypes of plants transformed with an empty vector that contains an RFP tag (WT) and six overexpression lines (RNAi-1, 5, and 8 without ABA treatment and RNAi-2, 6, and 9 with ABA treatment). **b** NBT staining dye in the leaves of transgenic lines with or without ABA treatment. **c**–**e** MDA content, relative water content, and chlorophyll content of transgenic lines with or without ABA treatment. **f** Expression of four ABA-responsive genes, *MdRD29A*, *MdRAB18*, *MdEM1*, and *MdEM6*, in transgenic lines with or without ABA treatment

ABA perception and signaling are major factors limiting apple production. To test whether MdWRKY31 was involved in the response to ABA in apple, *MdWRKY31* was first fused to the expression vector PRI-GFP along with the strong *35S* promoter upstream. The *35S::MdWRKY31-GFP* construct was then transformed into the roots of ‘Royal Gala’ seedlings using the *Agrobacterium rhizogenes*-mediated transformation method. Second, the specific fragment of MdWRKY31 fused into the expression vector pK7GWIWG2 along with an ERFP in its C-terminal region, after which the vector was transformed into the roots of ‘Royal Gala’ seedlings via the same transformation method. We then examined the fluorescence intensity and expression pattern of *MdWRKY31*. The results showed that the expression of *MdWRKY31* was upregulated in *MdWRKY31*-overexpressing apple roots (Fig. 6a, b) but noticeably downregulated in *MdWRKY31* RNAi-transformed roots (Fig. 6c, d). The wild type and three transgenic lines with different *MdWRKY31* expression levels were subsequently treated with 100 μM ABA for 10 days. We evaluated the phenotypes of the WT and *MdWRKY31* overexpressing/RNAi lines and found that the MdWRKY31 OE seedlings exhibited more sensitivity to ABA than did the wild type; by contrast, downregulating *MdWRKY31* expression reduced plant ABA sensitivity (Figs. 7a and 8a). Tetranitroblue tetrazolium chloride (NBT) staining indicated that *MdWRKY31* overexpression produced higher superoxide ion (O^2−^) than did the WT, and *MdWRKY31* RNAi lines accumulated less O^2−^ than did the WT (Figs. 7b and 8b). In addition, three *MdWRKY31*-overexpressing lines contained more MDA, lower RWC, and less chlorophyll than did the WT controls (Fig. 7c–e). These related physiological indicators of the ABA response were also detected in the leaves of *MdWRKY31* RNAi lines; the MdWRKY31 RNAi seedlings accumulated less MDA and more chlorophyll and had a higher RWC than did the WT (Fig. 8c–e). *MdWRKY31* transgenic apple roots exhibited great differences in ABA resistance; therefore, four ABA-responsive genes (*MdAD29A*, *MdRAB18*, *MdEM1*, and *MdEM6*) were measured. The results suggest that the expression of three of those genes (*MdRAB18*, *MdEM1*, and *MdEM6*) had varying degrees of increase in the *MdWRKY31*-overexpressing lines compared to the WT controls (Fig. 7f). Interestingly, compared to the WT controls, *MdWRKY31*-repressing lines (RNAi-6 and RNAi-9) also exhibited slightly higher expression of the abovementioned genes (*MdRAB18*, *MdEM1*, and *MdEM6*) (Fig. 8f). These results indicate that MdWRKY31 regulates ABA sensitivity in apple roots.

To confirm the function of *MdWRKY31* in apple, we transformed *35S::MdWRKY31* to apple calli and generated six *MdWRKY31* transgenic lines that present different *MdWRKY31* expression levels (Fig. S2a). *MdWRKY31* protein levels in the six transgenic lines were examined using an anti-GFP antibody. We found detectable MdWRKY31 proteins in all six transgenic calli (Fig. S2b). Next, three independent lines (OE-1, OE-2, and OE-3) were selected for subsequent experiments. Apple calli were treated with 50, 100, and 150 μM ABA. Evaluation of the phenotypes and fresh weight revealed that ABA at different concentrations inhibited apple callus growth and that the degree of inhibition was more obvious in the transgenic calli than in the WT calli (Fig. S2c, d). These results further confirm that MdWRKY31 acts as a positive regulator in the ABA signaling pathway in apple.

### MdWRKY31 represses the transcription of *MdRAV1* by binding directly to its promoter in apple

**Fig. 9:**
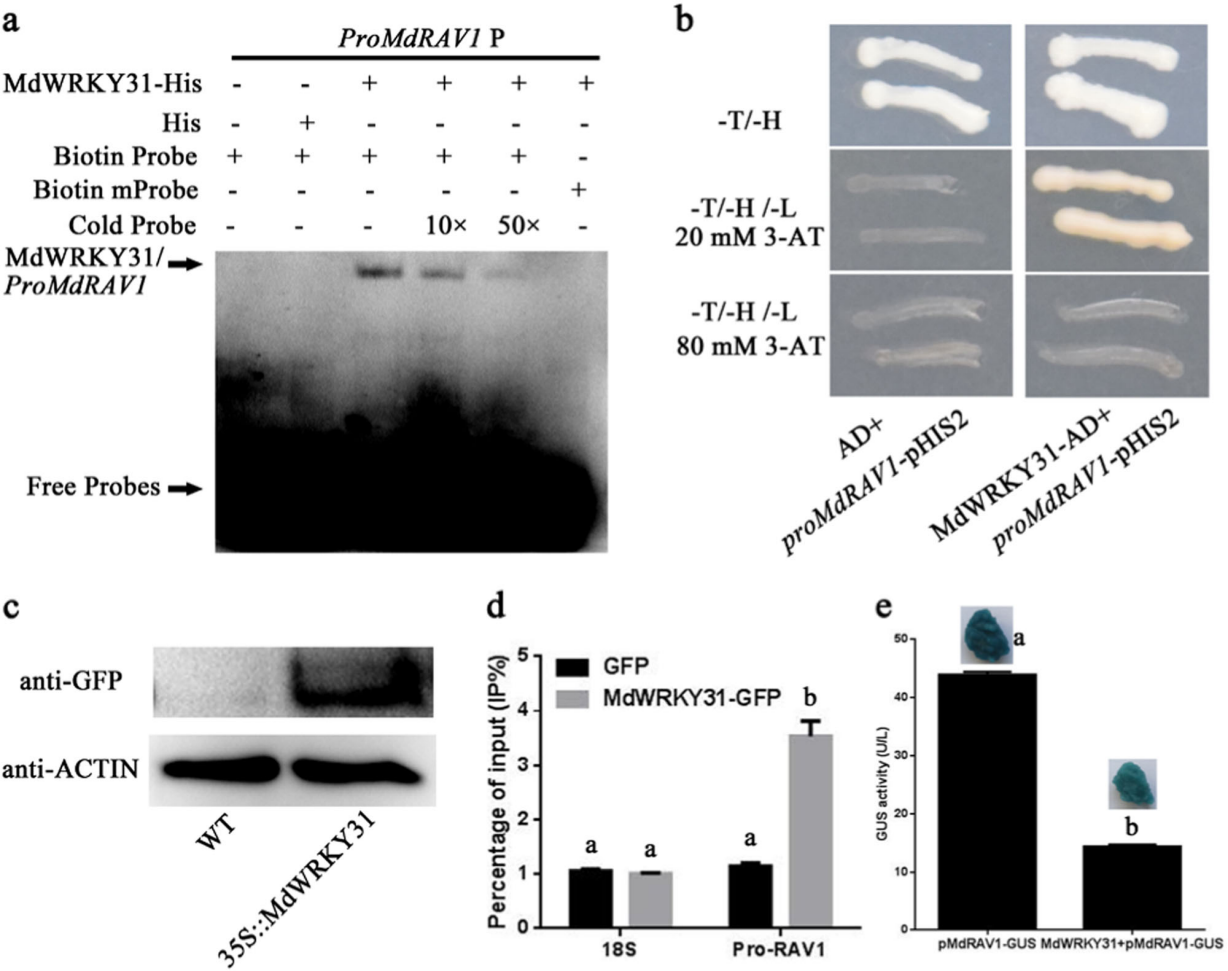
Direct repression of MdWRKY31 on the *MdRAV1* promoter. **a** EMSA of MdWRKY31 binding to the *MdRAV1* (MD16G1047700) promoter in vitro. Each biotin-labeled DNA probe was incubated with MdWRKY31-His protein. Mutation probes of P had the mutated W-box (TTGACA was replaced with TACGTC). **b** Interaction of MdWRKY31 with the promoter fragment of *MdRAV1* in a yeast one-hybrid assay. The constructs were cotransformed into yeast Y187. The yeast was cultured on a culture medium devoid of tryptophan and histidine (SD/-T/-H) and then screened for lack of tryptophan, leucine, and histidine (SD/-T/-L/-H) with 20 and 80 mM 3-amino-1,2,4-triazole (3-AT). **c** Immunoblot analysis of the MdWRKY31-GFP protein. Wild-type and *MdWRKY31*-overexpressing transgenic calli with a GFP fusion tag were harvested and evaluated for immunoblot analysis using an anti-GFP antibody. The loading control was anti-ACTIN. **d** ChIP-qPCR assay of MdWRKY31 binding to the *MdRAV1* promoter in vivo. ChIP-PCR revealed enriched DNA fragments of *MdWRKY31*-overexpressing calli (*MdWRKY31-GFP*) and calli transformed with an empty vector (*GFP*). The 18S served as the loading control. **e** GUS activity in transgenic calli as indicated. Quantitative statistics of GUS activity and GUS staining in pMdRAV1-GUS transgenic apple calli treated with or without *MdWRKY31* and transiently transformed

To explore the mechanism of MdWRKY31-regulated ABA resistance, related genes associated with ABA signaling were monitored in WT and *MdWRKY31*-overexpressing apple roots (Fig. S3). Many genes were upregulated and downregulated to varying degrees in *MdWRKY31* OE roots compared to WT roots. Interestingly, the expression of two *MdRAV* genes, *MdRAV1* (MD16G1047700) and *MdRAV2* (MD13G1046100), was clearly suppressed in the *MdWRKY31* OE roots. The promoters of the *MdRAV1* and *MdRAV2* genes were analyzed, and one W-box motif, a WRKY binding motif, was identified in both promoter sequences (Table S3). Therefore, we deduced that MdWRKY31 binds to the promoters of *MdRAV*s. First, an electrophoretic mobility shift assays (EMSA) was conducted to detect the binding of MdWRKY31 to the promoter regions of *MdRAV1* or *MdRAV2*. The results indicated that MdWRKY31 binds directly to the promoter of *MdRAV1* but not to the promoter of *MdRAV2* (Fig. S4a) in vitro (Fig. 9a). When the TTGACC *cis*-element was replaced with the TACGTC element, the binding stopped (Fig. 9a), indicating specific binding. Yeast one-hybrid and chromatin immunoprecipitation (ChIP) experiments then confirmed the interaction between MdWRKY31 and the *MdRAV1* promoter (Fig. 9b–d).

These results demonstrate that MdWRKY31 binds to the promoter of *MdRAV1*. To further examine whether the transcriptional activity of *MdRAV1* was induced or suppressed by MdWRKY31, a GUS reporter gene was fused downstream from the *MdRAV1* promoter and labeled *pMdRAV1::GUS*. Additionally, we transformed *pMdRAV1::GUS* into apple calli and obtained *pMdRAV1::GUS*-overexpressing apple calli. *MdWRKY31* was then transiently transformed into *pMdRAV1:GUS* and *p1300-GN::GUS* transgenic calli. We compared the GUS activity of the two transgenic calli and found that, compared with the *GUS*-overexpressing calli, the transgenic calli containing MdWRKY31 and *pMdRAV1-GUS* repressed the transcriptional activity of GUS (Fig. 9e). In general, the repression of *MdRAV1* transcription in the *MdWRKY31* transgenic calli and GUS staining results demonstrated that *MdWRKY31* noticeably suppressed *MdRAV1* expression (Fig. S3; Fig. 9e). Based on these findings, MdWRKY31 binds directly to the promoter regions of *MdRAV1* to repress its expression.

### MdRAV1 directly binds to promoters of *MdABI*s to repress their expression

**Fig. 10:**
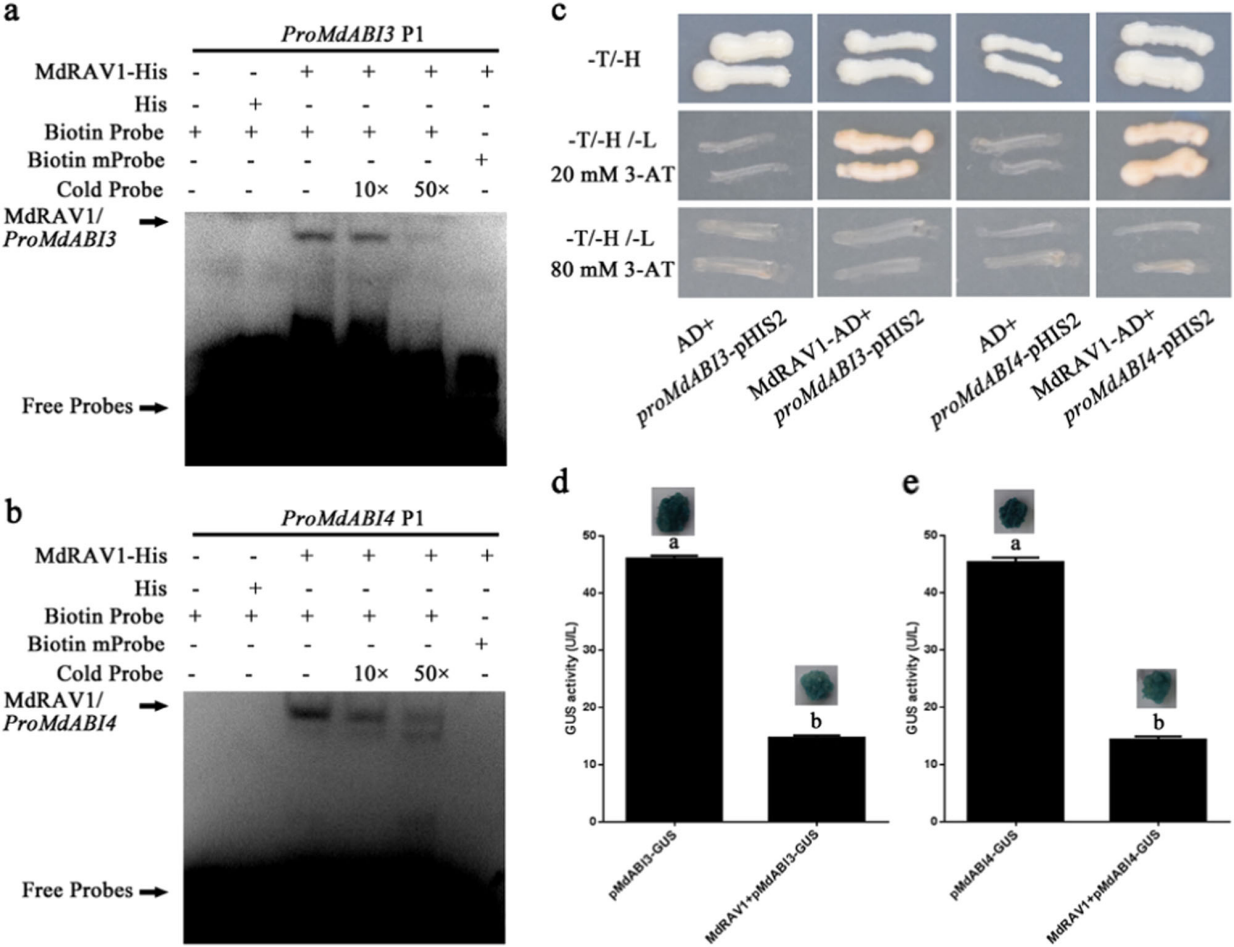
Direct repression of MdRAV1 on *MdABI* promoters. **a** EMSA of MdRAV1 binding to the *MdABI3* (*MD10G1169900*) promoter in vitro. Each biotin-labeled DNA probe was incubated with MdRAV1-His protein. Mutation probes of P1 had a mutated W-box (CAACA was replaced with TGGGG). **b** EMSA of MdRAV1 binding to the *MdABI4* (*MD07G1224400*) promoter in vitro. Each biotin-labeled DNA probe was incubated with MdRAV1-His protein. Mutation probes of P1 had a mutated element (CAACA was replaced with TGGGG). **c** Interaction of MdRAV1 with the promoter fragment of *MdABI3* and *MdABI4* in the yeast one-hybrid assay. The constructs were cotransformed into yeast Y187. The yeast was cultured on a culture medium devoid of tryptophan and histidine (SD/-T/-H) and then screened for lack of tryptophan, leucine, and histidine (SD/-T/-L/-H) with 20 and 80 mM of 3-amino-1,2,4-triazole (3-AT). **d**, **e** GUS activity in transgenic calli as indicated

As a transcription factor, AtRAV1 can bind to the promoters of *AtABI3*, *AtABI4*, and *AtABI5*^27^. On the basis of the expression levels of MdABIs, the expression of *MdABI3*, *MdABI4*, and *MdABI5* obviously increased in the *MdWRKY31* OE lines compared to the WT (Fig. S3). We analyzed the promoter regions of the three *MdABI* genes and found several conserved *cis*-acting elements (CAACA) in promoters of all three *MdABI* genes (Fig. S5). An EMSA assay was then carried out to detect interactions between MdRAV1 and the promoters of the *MdABI* genes. The results indicate that MdRAV1 could bind to CAACA at the P1 location of the promoters of *MdABI3* and *MdABI4* (Fig. 10a, b) but not *MdABI5* in vitro (Fig. S4b). CAACA was subsequently mutated to TGGGG; binding was not detected (Fig. 10a, b), indicating specific binding. Yeast one-hybrid and ChIP assays then demonstrated the interaction between MdRAV1 and the *MdABI3* and *MdABI4* promoters (Fig. 10c).

To verify the suppression or activation of MdRAV1 on *MdABI3* and *MdABI4*, the *MdABI3* and *MdABI4* promoters were fused to the *GUS* reporter gene downstream. We transformed *pMdABI3::GUS* and *pMdABI4::GUS* into apple calli and obtained *pMdABI3::GUS* and *pMdABI4::GUS* transgenic calli. *MdRAV1* was then transiently transformed into the *pMdABI3::GUS* and *pMdABI4::GUS* transgenic calli. GUS activities in different types of apple calli were detected, and the results show that MdRAV1 repressed the transcription activity of *pMdABI3::GUS* and *pMdABI4::GUS* (Fig. 10d). Therefore, MdRAV1 bound directly to the promoter regions of *MdABI3* and *MdABI4* to repress their expression.

## Discussion

### *MdWRKY31* can be induced by ABA

ABA is one of the most common factors affecting apple quality and yield. In ABA signaling, the rapid accumulation of ABA in response to drought protects plants against damage^29^. Many studies have shown that WRKYs play vital roles in ABA signaling and drought^4,24,30^. Transcriptome analysis has been indicated to be an effective method in biological research in recent years^31–33^. We used RNA-seq analysis to identify possible MdWRKYs that function in the ABA signaling pathway. Fortunately, one apple WRKY TF, *MdWRKY31*, was screened and found to be induced by ABA (Table S2). Further quantitative analysis revealed that ABA and PEG4000 (for simulating drought) significantly induced the expression of *MdWRKY31*, suggesting that *MdWRKY31* may be related to ABA signaling and drought. We also found that treatment with NaCl and 4 °C repressed *MdWRKY31* expression, implying that *MdWRKY31* is involved in various abiotic stresses.

### WRKY31 is conserved in different plant species

WRKY proteins are often classified into groups according to the number of WRKY domains and the features of their zinc-finger-like motif^15^. MdWRKY31 belongs to Group II and is homologous to AtWRKY42, AtWRKY47, AtWRKY31, and AtWRKY6 in *Arabidopsis*. In soybean, GmWRKY31 is involved in SA-mediated immune responses by binding the *cis*-acting W-box element of the *GmSAGT1* gene^34^. In rice, WRKY55/WRKY31 positively regulates resistance to *Magnaporthe oryzae*^35^. The WRKY domain and phylogenetic tree analysis of WRKY31s implied that the function of WRKY31 could be conserved among different plant species.

### MdWRKY31 localizes to the cell nucleus

Most transcription factors are located in the nucleus to perform their functions. OsWRKY67 positively regulates resistance against blast and bacterial blight and is ubiquitously expressed and sublocalized in the nucleus^36^. In grapevine, VaWRKY14 (involved in drought resistance) is localized to the nucleus^37^. A cucumber WRKY transcription factor, CsWRKY46, found exclusively in the nucleus, increases resistance to cold^38^. The location of MdWRKY31 indicates that it also is present in the cell nucleus, suggesting that MdWRKY31 potentially performs its function there.

### *MdWRKY31* positively regulates ABA signaling

Many reports have found that WRKYs are involved in ABA signaling. In Arabidopsis, WRKY41 controls seed dormancy by directly regulating *ABI3* expression; WRKY40 directly represses the expression of the *ABI5* ABA-responsive gene^19,22^. WRKY6 positively regulates ABA signaling by directly inhibiting the expression of *RAV1*^24^. FvWRKY42 increases ABA sensitivity and improves osmotic stress resistance in strawberry^30^. In cotton, GhWRKY6-like improves salt tolerance by activating the ABA signaling pathway^39^. We found that ectopic expression of *MdWRKY31* in Arabidopsis and tobacco and its homologous expression in apple calli and roots can increase sensitivity to ABA; thus, MdWRKY31 may positively mediate ABA signaling in plants.

It is difficult to obtain transgenic apple lines with stable expression because of the immaturity of the apple transformation system and its characteristics as a perennial woody plant. However, *Agrobacterium rhizogenes*-mediated transformation into apple roots renders gene function identification possible^40^. In this study, the function of *MdWRKY31* was confirmed in apple roots. Phenotypes of apple roots overexpressing and repressing *MdWRKY31* indicated that MdWRKY31 functions as a positive regulator in the ABA signaling pathway. The ABA-responsive genes *RAB18* and *RD29B* in *Arabidopsis* have always been used as indicators of the ABA signaling pathway^41,42^. *EM1*, *EM6*, and *RAB18* are three representative ABA-responsive genes. Analysis of the changes in the expression of these genes in transgenic *MdWRKY31* overexpression or suppression apple roots indicated that relevant research from *Arabidopsis* is applicable to other species, such as apple. The phenotypes of different *MdWRKY31* transgenic materials (Arabidopsis, tobacco, apple calli, and apple roots) suggested that the function of *MdWRKY31* in the ABA signaling pathway is conserved beyond plant species.

O^2−^, a form of ROS (reactive oxygen), is a toxic molecule that can cause oxidative damage to proteins, DNA, and lipids^43^. ABA induces the production of ROS^44^. In this study, *MdWRKY31* overexpression in transgenic apple plants promoted the accumulation of O^2−^ in response to ABA. The release of reactive oxygen species can cause chlorophyll degradation, programmed cell death, and MDA accumulation^45,46^. *MdWRKY31* overexpression in transgenic plants subsequently produced a higher MDA content and less chlorophyll and RWC in those plants than in the WT controls in response to ABA treatment.

### MdWRKY31 can bind to the promoters of MdRAV1, which interact with the promoters of *MdABI3* and *MdABI4*

Many protein kinases and transcription factors are involved in ABA signaling. ABI3, ABI4, and ABI5 mediate seed dormancy and seedling development^47–53^, and ABF2, ABF3, and ABF4 promote chlorophyll degradation and leaf senescence through ABA signaling^54^. SnRK2 kinases (SRK2D/SnRK2.2, SRK2E/SnRK2.6 and SRK2I/SnRK2.3 in Arabidopsis) play vital positive roles in ABA signaling downstream of the pyrabactin resistance1/PYR1-like/regulatory components of ABA receptor (PYR/PYL/RCAR) proteins^12,13,55,56^. The Arabidopsis RAV1 transcription factor provides plant insensitivity to ABA^27^. *MdWRKY31* overexpression alters the expression of these genes involved in ABA signaling. The repression of *MdRAV1* and increased expression of *ABF*s, *ABI*s, and *SnRK*s indicated that MdWRKY31 may be a positive regulator in ABA signaling. Studies have shown that AtWRKY42, which clustered in a group together with MdWRKY31, often functions as a transcriptional repressor^57^. Therefore, we proposed that MdWRKY31 also acted as a repressor of downstream genes; the *MdRAV* genes, whose expression was downregulated in the *MdWRKY31*-overexpressing plants, were selected for further investigation of their interaction. Direct binding of MdWRKY31 to the promoter regions of *MdRAV1* and the binding of MdRAV1 to the promoters of *MdABI3* and *MdABI4* subsequently revealed that MdWRKY31 participated in ABA signaling by interacting directly with ABA-related genes to repress their transcription.

### Conclusions

In conclusion, we identified the WRKY TF *MdWRKY31* in apple, which was significantly induced by ABA. Compared with control plants, *MdWRKY31* transgenic plant materials, including *Arabidopsis*, tobacco, and apple, exhibited greater ABA sensitivity, indicating that MdWRKY31 is a positive regulator in the ABA signaling pathway. Furthermore, we found that MdWRKY31 repressed the transcription of the *MdRAV1* gene by binding directly to promoter region of *MdRAV1*. The results further revealed that MdRAV1 bound to the promoters of *MdABI3* and *MdABI4* and inhibited their expression. Our findings identified the function of *MdWRKY31* in plants and the regulatory mechanism of MdWRKY31 to ABA sensitivity, which is useful in comprehending the complex TF-regulated network, and provide a potential gene for apple cultivar improvement. Rootstock improvement is also practical for apple, which is a grafted crop species. Apple root transformation may be a possible way to alter rootstock characteristics.

## Materials and methods

### Plant materials and experimental treatments

The ‘Orin’ callus cultivar was cultured on MS medium containing 0.5 mg/L 2,4-dichlorophenoxyacetic acid (2,4-D) and 1.5 mg/L 6-benzylaminopurine (6-BA) at 26 °C in the dark. Tissue cultures of the ‘Royal Gala’ cultivar in vitro were grown on MS medium supplemented with 1.5 mg/L 6-BA and 0.2 mg/L IAA at 26 °C under a 16-h light/8-h dark photoperiod. The roots of ‘Royal Gala’ tissue-cultured seedlings were treated with water (control), 100 μmol/L ABA, 4 °C temperature (low temperature), 100 μmol/L NaCl, or PEG4000 for 0, 1, 3, 6, 12, and 24 h. The samples were then quickly frozen in liquid nitrogen and stored in a refrigerator at −80 °C.

*Nicotiana benthamiana* (Ben), wild-type *Arabidopsis* (COL), and transgenic *Nicotiana benthamiana* and *Arabidopsis* were screened by MS media with 60 mg/L kanamycin. Polymerase chain reaction (PCR) detected positive transgenic plants. After continuous screening for three generations, T3 homozygous plants were obtained and used for phenotypic experiments. *Arabidopsis* seeds were sown on MS medium with or without ABA for the germination experiments. Images were taken after 8 days of growth, after which the germination rate was analyzed. Four days after being sown, seedlings without ABA treatment were transferred to 1/2-strength MS media or 1/2-strength MS media with 10 μM ABA. These culture dishes were placed in a growth chamber at 25 °C under a 16-h photoperiod. The root length of the seedlings was evaluated after 7 days, and the average value of 40 plants was counted. Transgenic apple lines were transferred to a nutrient-rich potting medium that mixed with vermiculite (1:1) and treated with 100 μM ABA or no ABA for 10 days. Plant leaves were then used to analyze ABA-related indices, including the NBT dyeing, MDA content, relative water content, chlorophyll content, and relevant ABA-responsive genes.

### Subcellular localization analysis

To explore the subcellular localization of MdWRKY31, the open reading frame (ORF) of *MdWRKY31* was amplified from ‘Royal Gala’ apple tissue culture seedlings using PCR in conjunction with MdWRKY31-F and MdWRKY31-R as primers (Table S1). Based on *MdWRKY31* sequences, the Gateway system was used to insert the *MdWRKY31* ORF into the pENTR^TM^ Directional TOPO vector with MdWRKY31-TOPO upstream and downstream primers. The recombination reaction (LR) was used to insert the *MdWRKY31* gene into the PAL1107 vector to construct a *MdWRKY31-RFP* vector for subcellular localization.

Bacterial solutions of MdWRKY31-RFP and P19 were cultured for 12 h. After centrifugation, the *Agrobacterium* liquid was suspended in an MMA liquid medium and mixed in a 1:1 proportion of MdWRKY31-RFP:P19 for 4–5 h. A 500 μl suspension liquid was injected into the leaves of *Nicotiana benthamiana*. Images were taken with a two-photon laser confocal microscope (Carl Zeiss;German) at 3–4 days after the plants were transferred to a growth chamber with a temperature of 25 °C and a 16-h photoperiod.

### Construction of vectors and obtained transgenic lines

To construct *MdWRKY31* overexpression vectors, the ORF of *MdWRKY31* was digested with SalI/KpnI and cloned into GFP plant transformation vectors downstream of the CaMV *35S* promoter. The *MdWRKY31-GFP* vector was then transformed into *Agrobacterium rhizogenes pRi2659* (Weidi Biotechnology, Shanghai, China). According to the *Agrobacterium* conversion method, the *MdWRKY31-GFP* vector was successfully genetically introduced into the roots of apple plants.

A specific sequence of *MdWRKY31* via primers MdWRKY31-FR-F and MdWRKY31-FR-R was inserted into the pENTR^TM^ Directional TOPO vector. The LR was used to insert the specific fragment of *MdWRKY31* into the pK7GWIWG2 vector to construct a *MdWRKY31-RFP* vector. The resulting *MdWRKY31-RFP* vector was genetically introduced into apple plant roots according to *Agrobacterium rhizogenes* MSU44-mediated transformation as described by Ma et al.^40^. The primers used in this paper are detailed in Table S1.

### Analysis of gene expression

TRIzol reagent (Invitrogen, Carlsbad, CA) was used to extract the total RNA from ‘Royal Gala’ tissues (calli and seedlings), *Arabidopsis*, and *Nicotiana benthamiana*. cDNA synthesis was executed with a PrimeScript^TM^ RT reagent kit (TaKaRa, Dalian, China). Quantitative real-time PCR (qRT-PCR) was used to test the *MdWRKY31* expression level in response to ABA, NaCl, 4 °C, and PEG4000. In different transgenic *Arabidopsis*, *Nicotiana benthamiana*, and apple tissues, *MdActin* (GenBank accession number CN938024) was used as the reference gene.

For qRT-PCR, mixed solutions were executed with iQ SYBR Green Supermix in an iCycler iQ5 system (Bio-Rad, Hercules, CA, USA) according to the manufacturer’s instructions. Analyses of specific mRNA levels were performed using relative quantification via the cycle threshold (Ct) 2-ΔΔCt method. Each assay was carried out for three biological replicates. PCR profiles were determined on the basis of the following protocol: preincubation at 95 °C for 5 min; 30 cycles of 95 °C (30 s), 58 °C (30 s), and 72 °C (30 s); and a 72 °C (5 min) final extension. The corresponding primers used are listed in Table S1.

### Determination of the relative water content and chlorophyll content

The relative water content (RWC) was determined based on the method described by Ma et al.^40^ as follows: RWC = (fresh weight – dry weight)/(rehydrated weight – dry weight).

Similarly, the chlorophyll content was measured according to the method described by An et al.^58^ as follows:

C_a_ = 13.95D_665_ − 6.88D_649_

C_b_ = 24.96D_649_ − 6.88D_665_

Chlorophyll content = C_a_ + C_b_

### MDA content and NBT staining

As described by Ma et al.^40^, the absorbance at 450, 532, and 600 nm was measured with an UV/vis spectrophotometer (UV‐2450). MDA levels were calculated as follows: MDA content (mmol/g FW) ¼[6.542*(OD532−OD600)−0.559*OD450] (mmol/L)*V (ml)/fresh weight (g FW).

Leaves soaked in NBT staining buffer (0.5 mg/ml) were vacuum treated for 20 min for better dyeing. The samples were then stained for 8 h in darkness at 28 °C and then boiled for 5 min with ethanol:lactic acid:glycerin (3:1:1) in a fixative solution until the chlorophyll was removed and the samples cooled. Anhydrous alcohol was then added prior to observations.

### GUS analysis

Apple calli were used for transient expression assays. Wild-type promoters of *MdRAV1*, *MdABI3*, and *MdABI4* were cloned into *p1300-GN*, which was fused to the *GUS* reporter gene. The constructed *pMdRAV1::GUS* plasmids (the *pMdABI3::GUS* plasmid and *pMdABI4::GUS* plasmid) were transformed into apple calli via the *Agrobacterium*-mediated method. *35S::MdWRKY31* (*35S::MdRAV1*) was then cotransformed into the *pMdRAV1::GUS* (*pMdABI3::GUS* and *pMdABI4::GUS*) and *p1300-GN::GUS* transgenic calli. Finally, histochemical staining was conducted to measure the GUS activity in the treated calli using the method described by Zhao et al. (2016)^59^.

### Chromatin immunoprecipitation (ChIP) qPCR analysis

The *35::MdWRKY31::GFP* and *35::GFP* transgenic calli were used for a ChIP assay. An anti-GFP antibody (Beyotime, Haimen, China) was applied to ChIP-qPCR as described by Hu et al.^60^. Immunoprecipitated samples were used as templates for the qPCR assay with primers listed in Table S1.

### Electrophoretic mobility shift assays (EMSAs)

An EMSA was performed as described by Xie et al.^61^. *MdWRKY31* and *MdRAV1* were cloned into the expression vector *pET-32a*(+). The MdWRKY31-HIS and MdRAV1-HIS recombinant proteins were expressed in *Escherichia coli* strain BL21 and purified using a His Microspin Purification kit (Tiangen, Beijing, China). The oligonucleotide probes of the *MdRAV1*, *MdABI3*, and *MdABI4* promoters were labeled by a company (Sangon Biotech, Beijing, China). The binding specificity was determined by measuring its competition with excess unlabeled oligonucleotides. The primers used are listed in Table S1.

### Statistical analysis

Appropriate methods using R (3.0.2) with the R Commander package were employed to analyze three parallel experiments statistically. Mean differences between the bars are significant at the *P*_0.05_ level for different letters but not significant at the *P*_0.05_ level for the same letters.

## Supplementary information

W Fig. S1 qRT-PCR determination of MdWRKY31 transgenic Nicotiana benthamiana

Fig. S2 MdWRKY31 confers increased ABA sensitivity in apple calli

Fig. S3 Relative expression of ABA-related genes in ABA signal in MdWRKY31 transgenic apple roots

Fig. S4 The EMSA assays of MdWRKY31 on MdRAV2 (MD13G1046100) promoter and MdRAV1 on MdABI5 (MD12G1034900) promoter

Fig. S5 Schematic representation of MdABI3 (MD10G1169900) and MdABI4 (MD07G1224400) locus

Supplement table 1 List of primers used in this research

Supplement table 3. MdRAV1-1 (MD13G1046100) and MdRAV1-2 (MD16G1047700) promoter cis-acting element analysis

## References

[1] Cutler SR, Rodriguez PL, Finkelstein RR, Abrams SR (2010). Abscisic acid: emergence of a core signaling network. Annu. Rev. Plant Biol..

[2] Raghavendra AS, Gonugunta VK, Christmann A, Grill E (2010). ABA perception and signalling. Trends Plant Sci..

[3] Weiner JJ, Peterson FC, Volkman BF, Cutler SR (2010). Structural and functional insights into core ABA signaling. Curr. Opin. Plant Biol..

[4] Wang S (2018). Abscisic acid is involved in aromatic ester biosynthesis related with ethylene in green apples. J. Plant Physiol..

[5] Pan QH (2005). Abscisic acid activates acid invertases in developing grape berry. Physiol. Plant..

[6] Assmann SM (1994). Ins and outs of guard cell ABA receptors. Plant Cell.

[7] Finkelstein RR, Lynch TJ (2000). The *Arabidopsis* abscisic acid response gene *ABI5* encodes a basic leucine zipper transcription factor. Plant Cell.

[8] Verslues PE, Zhu JK (2007). New developments in abscisic acid perception and metabolism. Curr. Opin. Plant Biol..

[9] Liu X (2007). AG protein coupled receptor is a plasma membrane receptor for the plant hormone abscisic acid. Science.

[10] Johnston CA (2007). Comment on a G protein coupled receptor is a plasma membrane receptor for the plant hormone abscisic acid. Science.

[11] Pandey S, Nelson DC, Assmann SM (2009). Two novel GPCR-type G proteins are abscisic acid receptors in *Arabidopsis*. Cell.

[12] Ma Y (2009). Regulators of PP2C phosphatase activity function as abscisic acid sensors. Science.

[13] Park SY (2009). Abscisic acid inhibits type 2C protein phosphatases via the PYR/PYL family of START proteins. Science.

[14] Fujii H (2009). In vitro reconstitution of an abscisic acid signalling pathway. Nature.

[15] Eulgem T, Rushton PJ, Robatzek S, Somssich IE (2000). The WRKY superfamily of plant transcription factors. Trends Plant Sci..

[16] Rushton PJ, Somssich IE, Ringler P, Shen QJ (2010). WRKY transcription factors. Trends Plant Sci..

[17] Finkelstein RR, Wang ML, Lynch TJ, Rao S, Goodman HM (1998). The *Arabidopsis* abscisic acid response locus *ABI4* encodes an APETALA2 domain protein. Plant Cell.

[18] Lopez-Molina L, Chua NH (2000). A null mutation in a bZIP factor confers ABA-insensitivity in *Arabidopsis thaliana*. Plant Cell Physiol..

[19] Shang Y (2010). The Mg-chelatase H subunit of *Arabidopsis* antagonizes a group of WRKY transcription repressors to relieve ABA-responsive genes of inhibition. Plant Cell.

[20] Ren XZ (2010). ABO3, a WRKY transcription factor, mediates plant responses to abscisic acid and drought tolerance in Arabidopsis. Plant J..

[21] Jiang W, Yu D (2009). *Arabidopsis* WRKY2 transcription factor mediates seed germination and postgermination arrest of development by abscisic acid. BMC Plant Biol..

[22] Ding ZJ (2014). WRKY 41 controls *Arabidopsis* seed dormancy via direct regulation of ABI3 transcript levels not downstream of ABA. Plant J..

[23] Chen L, Zhang L, Li D, Wang F, Yu D (2013). WRKY8 transcription factor functions in the TMV-cg defense response by mediating both abscisic acid and ethylene signaling in *Arabidopsis*. Proc. Natl Acad. Sci. USA.

[24] Huang Y, Feng CZ, Ye Q, Wu WH, Chen YF (2016). *Arabidopsis* WRKY6 transcription factor acts as a positive regulator of abscisic acid signaling during seed germination and early seedling development. PLoS Genet..

[25] Hu YX, Wang YH, Liu XF, Li JY (2004). *Arabidopsis* RAV1 is down-regulated by brassinosteroid and may act as a negative regulator during plant development. Cell Res..

[26] Woo HR (2010). The RAV1 transcription factor positively regulates leaf senescence in *Arabidopsis*. J. Exp. Bot..

[27] Feng CZ (2014). *Arabidopsis* RAV1 transcription factor, phosphorylated by SnRK2 kinases, regulates the expression of *ABI3*, *ABI4*, and *ABI5* during seed germination and early seedling development. Plant J..

[28] Chen H (2010). Roles of Arabidopsis WRKY18, WRKY40 and WRKY60 transcription factors in plant responses to abscisic acid and abiotic stress. BMC Plant Boil..

[29] Lee SC, Luan S (2012). ABA signal transduction at the crossroad of biotic and abiotic stress responses. Plant Cell Environ..

[30] Wei W (2018). Ectopic expression of FvWRKY42, a WRKY transcription factor from the diploid woodland strawberry (*Fragaria vesca*), enhances resistance to powdery mildew, improves osmotic stress resistance, and increases abscisic acid sensitivity in *Arabidopsis*. Plant Sci..

[31] Morozova O, Hirst M, Marra MA (2009). Applications of new sequencing technologies for transcriptome analysis. Annu. Rev. Genom. Hum. Genet..

[32] Mutz KO, Heilkenbrinker A, Lönne M, Walter JG, Stahl F (2013). Transcriptome analysis using next-generation sequencing. Curr. Opin. Biotechnol..

[33] Sudhagar A, Kumar G, El-Matbouli M (2018). Transcriptome analysis based on RNA-seq in understanding pathogenic mechanisms of diseases and the immune system of fish: a comprehensive review. Int. J. Mol. Sci..

[34] Dong, H. et al. Transcriptome analysis of soybean WRKY TFs in response to Peronospora manshurica infection. *Genomics*. (2018). 10.1016/j.ygeno.2018.09.014. 10.1016/j.ygeno.2018.09.014 30267765

[35] Cheng H, Wang S (2014). The important player of rice-pathogen interactions: WRKY-type transcription factors. Sci. Sin. Vitae.

[36] Liu Q (2018). OsWRKY67 positively regulates blast and bacteria blight resistance by direct activation of *PR* genes in rice. BMC Plant Biol..

[37] Zhang LL (2018). Overexpression of *VaWRKY14* increases drought tolerance in *Arabidopsis* by modulating the expression of stress-related genes. Plant Cell Rep..

[38] Zhang Y (2016). CsWRKY46, a WRKY transcription factor from cucumber, confers cold resistance in transgenic-plant by regulating a set of cold-stress responsive genes in an ABA-dependent manner. Plant Physiol. Biochem..

[39] Ullah A, Sun H, Yang X, Zhang X (2018). A novel cotton WRKY gene, *GhWRKY6-like*, improves salt tolerance by activating the ABA signaling pathway and scavenging of reactive oxygen species. Physiol. Plant..

[40] Ma QJ (2017). An apple CIPK protein kinase targets a novel residue of AREB transcription factor for ABA-dependent phosphorylation. Plant Cell Environ..

[41] Kang JY, Choi HI, Im MY, Kim SY (2002). *Arabidopsis* basic leucine zipper proteins that mediate stress-responsive abscisic acid signaling. Plant Cell.

[42] Kim S, Kang JY, Cho DI, Park JH, Kim SY (2004). ABF2, an ABRE-binding bZIP factor, is an essential component of glucose signaling and its overexpression affects multiple stress tolerance. Plant J..

[43] Apel K, Hirt H (2004). Reactive oxygen species: metabolism, oxidative stress, and signal transduction. Annu. Rev. Plant Biol..

[44] Pei ZM (2000). Calcium channels activated by hydrogen peroxide mediate abscisic acid signalling in guard cells. Nature.

[45] Delledonne M, Zeier J, Marocco A, Lamb C (2001). Signal interactions between nitric oxide and reactive oxygen intermediates in the plant hypersensitive disease resistance response. Proc. Natl Acad. Sci. USA.

[46] Asada K (2006). Production and scavenging of reactive oxygen species in chloroplasts and their functions. Plant Physiol..

[47] Parcy F (1994). Regulation of gene expression programs during *Arabidopsis* seed development: roles of the *ABI3* locus and of endogenous abscisic acid. Plant Cell.

[48] Lopez‐Molina L, Mongrand S, McLachlin DT, Chait BT, Chua NH (2002). ABI5 acts downstream of ABI3 to execute an ABA‐dependent growth arrest during germination. Plant J..

[49] Shu K (2013). ABI4 regulates primary seed dormancy by regulating the biogenesis of abscisic acid and gibberellins in. Arab. PLoS Genet..

[50] Shu K (2018). ABI4 regulates the floral transition independently of ABI5 and ABI3. Mol. Biol. Rep..

[51] Zhang X, Garreton V, Chua NH (2005). The AIP2 E3 ligase acts as a novel negative regulator of ABA signaling by promoting ABI3 degradation. Genes Dev..

[52] Bossi F (2009). The *Arabidopsis* ABA‐INSENSITIVE (ABI) 4 factor acts as a central transcription activator of the expression of its own gene, and for the induction of *ABI5* and *SBE2. 2* genes during sugar signaling. Plant J..

[53] Lopez-Molina L, Mongrand S, Kinoshita N, Chua NH (2003). AFP is a novel negative regulator of ABA signaling that promotes ABI5 protein degradation. Genes Dev..

[54] Gao S (2016). ABF2, ABF3, and ABF4 promote ABA-mediated chlorophyll degradation and leaf senescence by transcriptional activation of chlorophyll catabolic genes and senescence-associated genes in *Arabidopsis*. Mol. Plant.

[55] Nishimura N (2009). Structural mechanism of abscisic acid binding and signaling by dimeric PYR1. Science.

[56] Santiago J (2009). The abscisic acid receptor PYR1 in complex with abscisic acid. Nature.

[57] Su T (2015). WRKY42 modulates phosphate homeostasis through regulating phosphate translocation and acquisition in Arabidopsis. Plant Physiol..

[58] An JP (2017). Ectopic expression of an apple cytochrome P450 gene *MdCYPM1* negatively regulates plant photomorphogenesis and stress response in *Arabidopsis*. Biochem. Bioph. Res. Co..

[59] Zhao Q (2016). Overexpression of *MdbHLH104* gene enhances the tolerance to iron deficiency in apple. Plant Biotechnol. J..

[60] Hu DG (2016). MdMYB1 regulates anthocyanin and malate accumulation by directly facilitating their transport into vacuoles in apples. Plant Physiol..

[61] Xie XB (2012). The bHLH transcription factor MdbHLH3 promotes anthocyanin accumulation and fruit colouration in response to low temperature in apples. Plant Cell Environ..

