# Supplementary material for: The MdWRKY31 transcription factor binds to the MdRAV1 promoter to mediate ABA sensitivity
Source: Hortic Res. 2019 Jun 1;6:66. doi: 10.1038/s41438-019-0147-1 (PMC6544635; doi:10.1038/s41438-019-0147-1)

**Fig. S1** **qRT-PCR determination of *MdWRKY31* transgenic *Nicotiana benthamiana*.** Wild-type tobacco was Ben. 35S::MdWRKY31 was *MdWRKY31* overexpressing transgenic tobacco. 1–5 represent different strains.


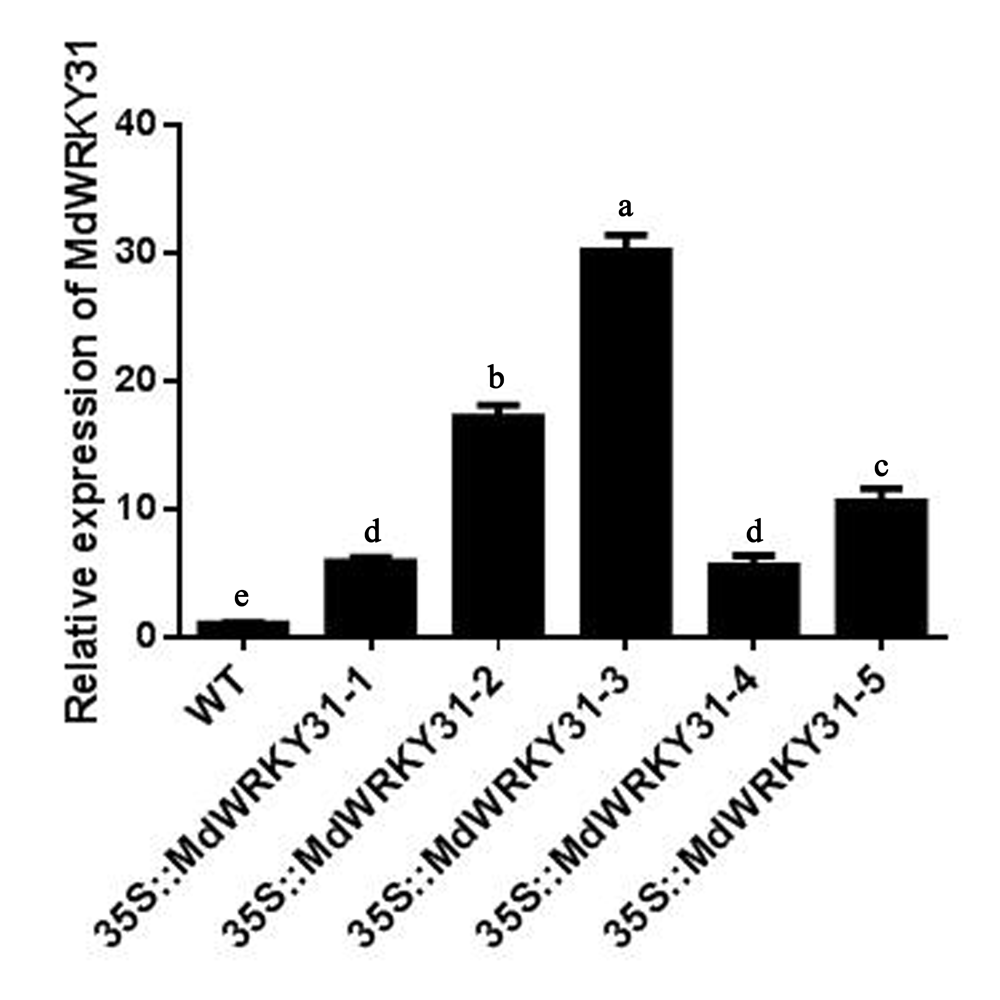

Supplement: Supplementary file 1 — W Fig. S1 qRT-PCR determination of MdWRKY31 transgenic Nicotiana benthamiana [file 41438_2019_147_MOESM1_ESM.doc]
