# Supplementary material for: The MdWRKY31 transcription factor binds to the MdRAV1 promoter to mediate ABA sensitivity
Source: Hortic Res. 2019 Jun 1;6:66. doi: 10.1038/s41438-019-0147-1 (PMC6544635; doi:10.1038/s41438-019-0147-1)

**Fig. S2** ***MdWRKY31* confers increased ABA sensitivity in apple calli.** **a** qRT-PCR detection of *MdWRKY31-OE* transgenic calli. WT represents wild-type calli. OE represents different *MdWRKY31-OE* transgenic lines. 1–6 represent different lines. **b** MdWRKY31 protein level in apple calli. MdWRKY31-GFP fusion protein was detected by immunoblot analysis using an anti-GFP antibody. The loading control was anti-ACTIN. **c** Phenotypes of different types of calli treated with or without ABA in medium. **d** Fresh weight of calli treated with or without ABA in medium. OE was *MdWRKY31*-overexpressing apple calli. 1–3 represent different strains.


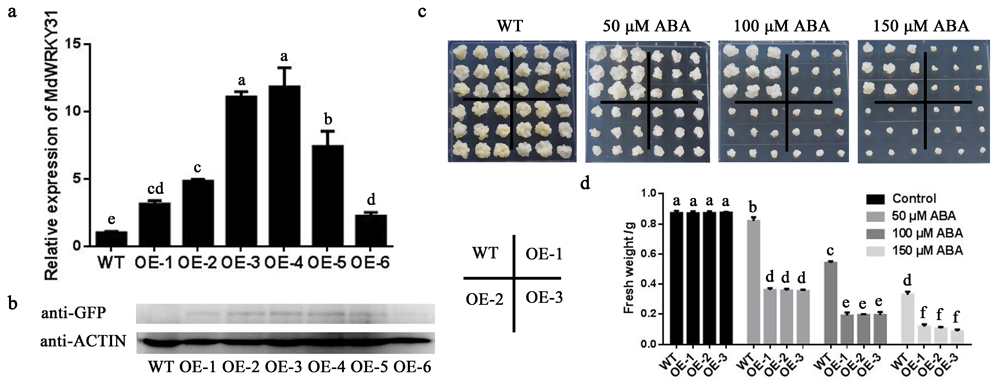

Supplement: Supplementary file 2 — Fig. S2 MdWRKY31 confers increased ABA sensitivity in apple calli [file 41438_2019_147_MOESM2_ESM.doc]
