# Supplementary material for: The MdWRKY31 transcription factor binds to the MdRAV1 promoter to mediate ABA sensitivity
Source: Hortic Res. 2019 Jun 1;6:66. doi: 10.1038/s41438-019-0147-1 (PMC6544635; doi:10.1038/s41438-019-0147-1)

**Fig. S3 Relative expression of ABA-related genes in ABA signal in *MdWRKY31* transgenic apple roots.** Wild-type was empty vector containing a GFP tag; OE was *MdWRKY31*-overexpressing lines. 1,4,7 represent different strains.


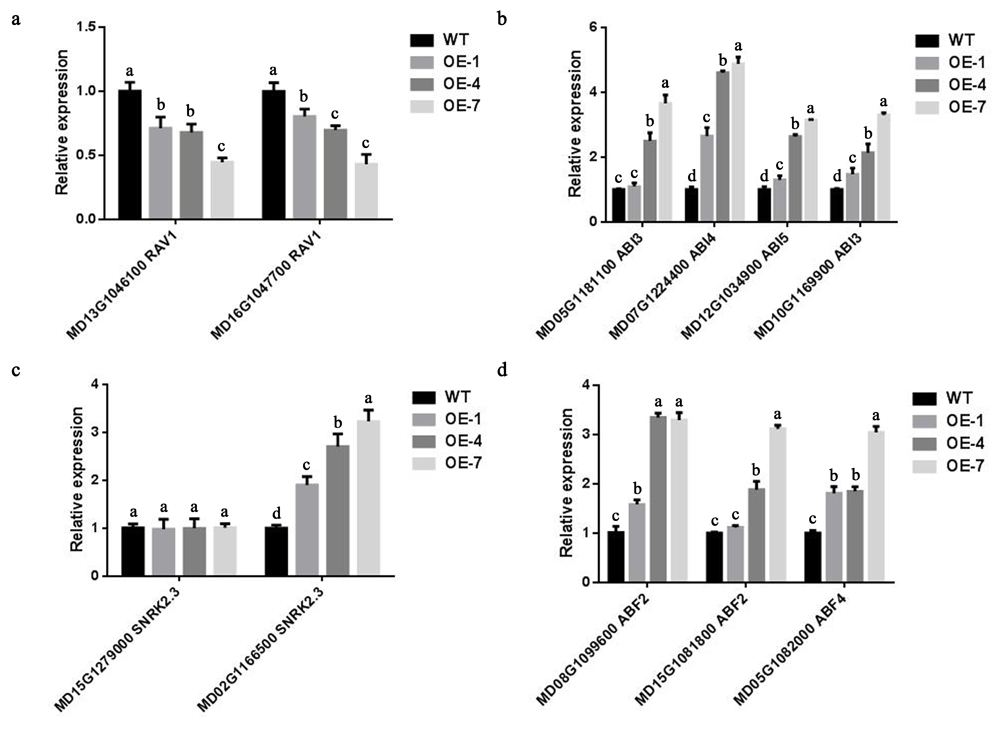

Supplement: Supplementary file 3 — Fig. S3 Relative expression of ABA-related genes in ABA signal in MdWRKY31 transgenic apple roots [file 41438_2019_147_MOESM3_ESM.doc]
