# Supplementary material for: The MdWRKY31 transcription factor binds to the MdRAV1 promoter to mediate ABA sensitivity
Source: Hortic Res. 2019 Jun 1;6:66. doi: 10.1038/s41438-019-0147-1 (PMC6544635; doi:10.1038/s41438-019-0147-1)
Supplement: Supplementary file 4 — Fig. S4 The EMSA assays of MdWRKY31 on MdRAV2 (MD13G1046100) promoter and MdRAV1 on MdABI5 (MD12G1034900) promoter [file 41438_2019_147_MOESM4_ESM.doc]

**Fig. S4 The EMSA assays of MdWRKY31 on *MdRAV2* (MD13G1046100) promoter and MdRAV1 on *MdABI5* (*MD12G1034900*) promoter.** **a** The binding of MdWRKY31 on *MdRAV2* (MD13G1046100) promoter in vitro. Each biotin-labeled DNA probe was incubated with MdWRKY31-His protein. Mutation probes of P had the mutated W-box (TTGACA was replaced by TACGTC). **b** EMSA of MdRAV1 binding to *MdABI5* (*MD12G1034900*) promoter in vitro. Each biotin-labeled DNA probe was incubated with MdRAV1-His protein. Mutation probes of P had mutated (CAACA was replaced by TGGGG).

**
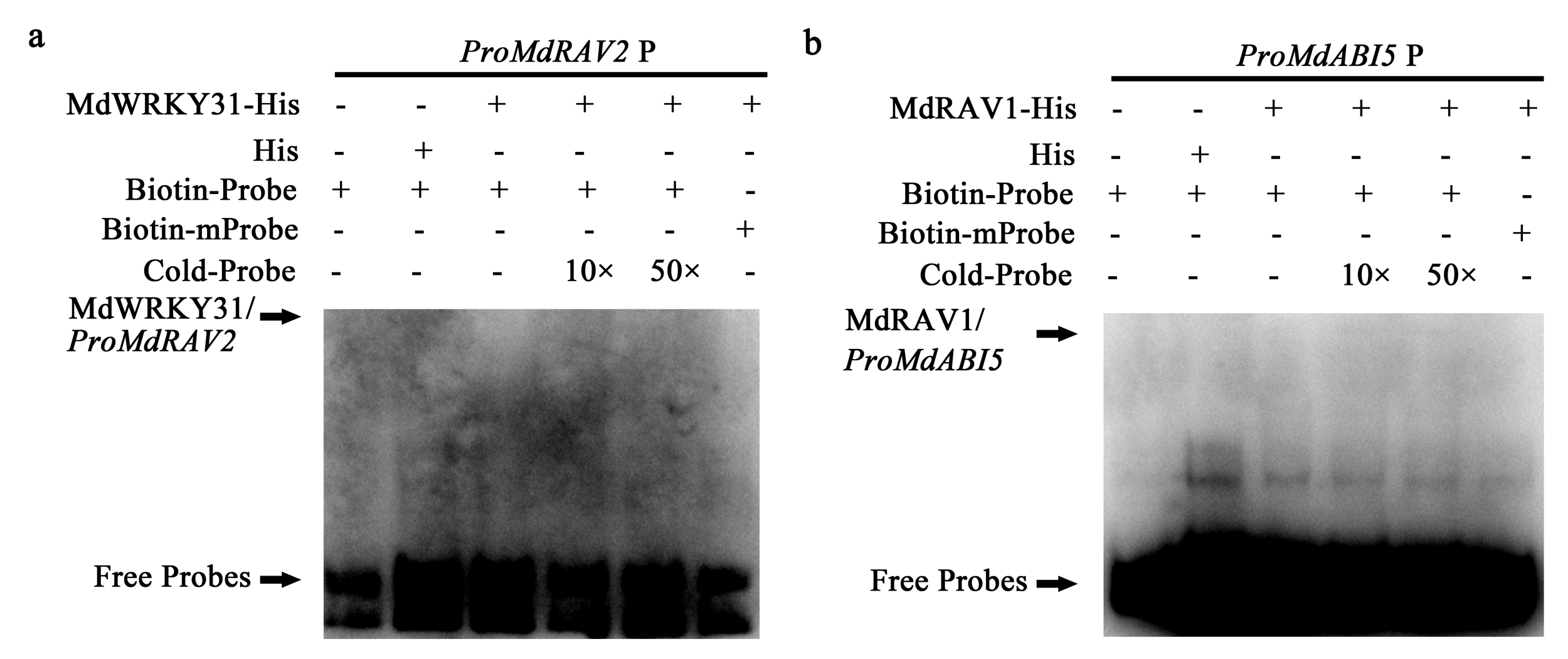
**
