# Supplementary material for: The MdWRKY31 transcription factor binds to the MdRAV1 promoter to mediate ABA sensitivity
Source: Hortic Res. 2019 Jun 1;6:66. doi: 10.1038/s41438-019-0147-1 (PMC6544635; doi:10.1038/s41438-019-0147-1)
Supplement: Supplementary file 5 — Fig. S5 Schematic representation of MdABI3 (MD10G1169900) and MdABI4 (MD07G1224400) locus [file 41438_2019_147_MOESM5_ESM.doc]

**Fig. S5 Schematic representation of *MdABI3* (MD10G1169900) and *MdABI4* (MD07G1224400) locus. a**, **b** *MdABI3* (MD10G1169900) and *MdABI4* (MD07G1224400) putative promoter were indicated by black line showing relative positions of CAACA motifs (gray lines), and transcribed sequence by black box (exon) and gray boxes (untranslated regions). P1, P2 and P3 represent CAACA motifs.

**
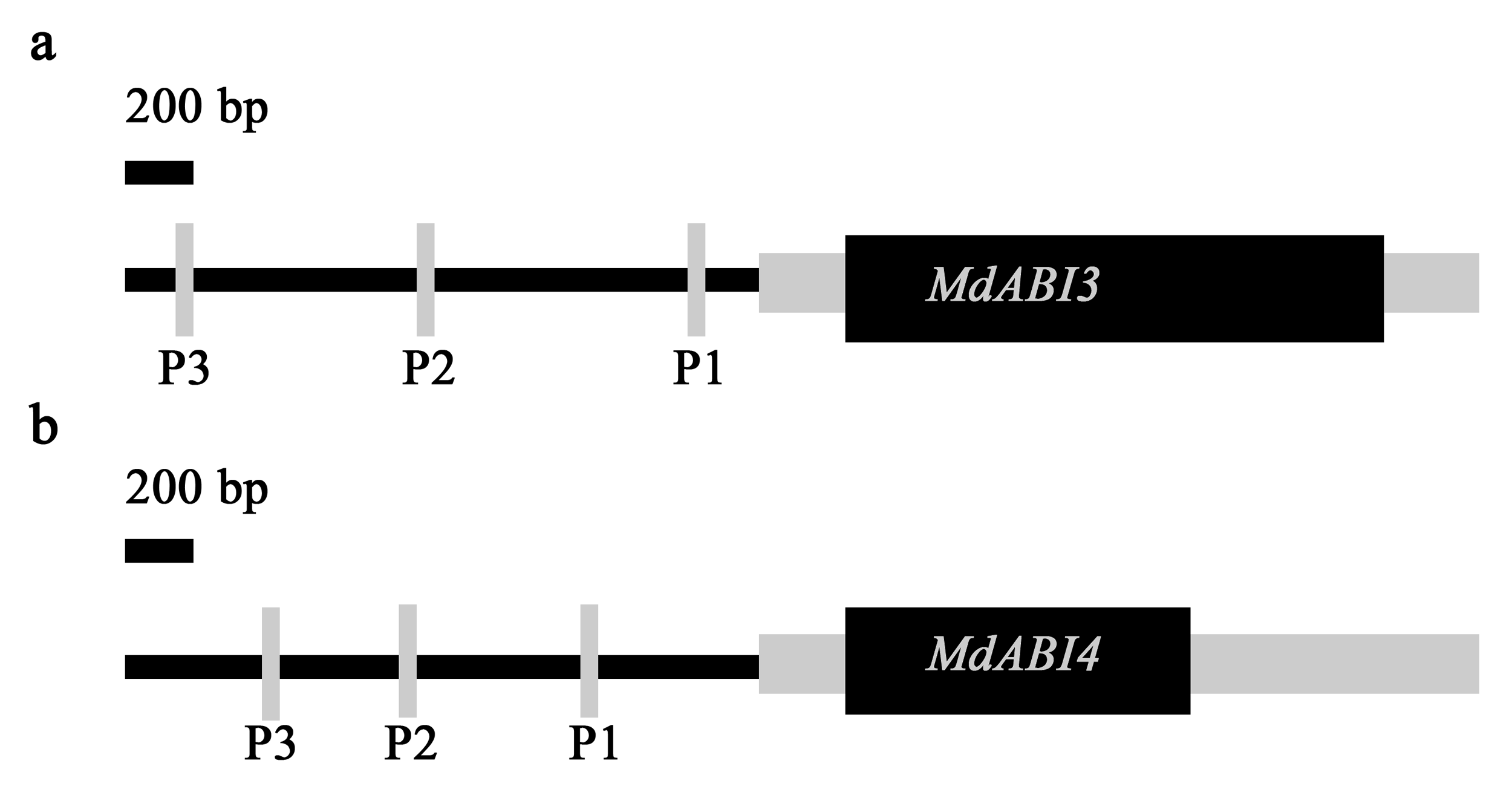
**
