# Supplementary material for: The MdWRKY31 transcription factor binds to the MdRAV1 promoter to mediate ABA sensitivity
Source: Hortic Res. 2019 Jun 1;6:66. doi: 10.1038/s41438-019-0147-1 (PMC6544635; doi:10.1038/s41438-019-0147-1)
Supplement: Supplementary file 6 — Supplement table 1 List of primers used in this research [file 41438_2019_147_MOESM6_ESM.doc]

**Supplement table 1 List of primers used in this research.**

| Gene name | Enzymes | Primer sequence(5'→3') |
| --- | --- | --- |
| MdWRKY31 |  | F: ATGGACAAAGGATGGGGGCTCACT  R: ATTTCCCGGGAAGCTGCTAATGTT |
| MdWRKY31(RT) |  | F: CCGTCCAATGACAACAACCG  R: TCCCATCGTCAACCATCGAC |
| MdWRKY31-FR-F |  | F: AAGGAGCCCTTCACCATGGACAAAGGATGG  R: GGCGCGCCCACCCTTAGTATTAACATCCAA |
| pET32a-MdWRKY31 | *EcoR*Ⅴ, *Sal*Ⅰ | F: GATATCATGGACAAAGGATGGGG  R: GTCGACTTAATTTCCCGGGAAG |
| pGAD424-MdWRKY31 | *Sal*Ⅰ, *Bgl* Ⅱ | F: GTCGACCTATGGACAAAGGATG  R: AGATCTTTAATTTCCCGGGAAG |
| MdWRKY31-TOPO |  | F: AAGGAGCCCTTCACCATGGACAAAGGATGGG  R:GGCGCGCCCACCCTTATTTCCCGGGAAGCTGC |
| MD16G1047700-pMdRAV1-1300-GUS | *BamH*Ⅰ, *Sma*Ⅰ | F: GGATCCTGTTCTTGTTTCGTTGATT  R: cccgggCTTTGTATTTTCTTTATCCT |
| pET32a-MD16G1047700-MdRAV1 | *BamH*Ⅰ, *EcoR*Ⅰ | F: GGATCC ATGGATGGAATAAGTA  R: GAATTC CAAAGCTCCGATGATC |
| pGAD424-MD16G1047700-MdRAV1 | *EcoR*Ⅰ, *BamH*Ⅰ | F: GAATTC ATGGATGGAATAAGTA  R: GGATCC CAAAGCTCCGATGATC |
| MD16G1047700-MdRAV1 (RT) |  | F: TACTTCTCTTACGGGAAGCGG  R: TTTGCAAGCGGAAGATGCAC |
| MD13G1046100-MdRAV2 (RT) |  | F: GGCTGGGTACCTTCAACGAA  R: TTGTTGCGCTTGCTCTGTTC |
| pGEX-4T-1- MD16G1047700-MdRAV1 | *BamH*Ⅰ, *EcoR*Ⅰ | F: GGATCC ATGGATGGAATAAGTA  R: GAATTC CAAAGCTCCGATGATC |
| PRI-GFP-MdWRKY31 | *Sal*Ⅰ, *Kpn*Ⅰ | F: gaattcATGGACAAAGGATGGGGGC  R: GGTACCATTTCCCGGGAAGCTGCTAA |
| MD05G1181100-ABI3(RT) |  | F: CAACGGCACACAAGAAAGAA  R: CCACCTCCACCACTACCACT |
| MD07G1224400-ABI4(RT) |  | F: GAAGCAGCACCAGATCATCA  R: GCCCTTGCACTTTCTGTTGT |
| MD12G1034900-ABI5(RT) |  | F: ACAATTTCCGAACGATCGAC  R: CAGAGAAGCATCGGTGACCT |
| MD10G1169900-ABI3(RT) |  | F: GGAGTGAAGGTACGGCAAGA  R: GCTGAAGAAGGTGACGATCC |
| MD15G1279000-SNRK2.3(RT) |  | F: CTCCTGCATACATTGCTCCA  R: GAAAGGATATGCTCCCACCA |
| MD02G1166500-SNRK2.3(RT) |  | F: AACCAGTTCGAAGAGCCTGA  R: TCCAGGTTGTCCGTCATGTA |
| MD08G1099600-ABF2(RT) |  | F: GCAGGCTTACACAACGGAAT  R: GGTCCTGCTGATTCTTCTGC |
| MD15G1081800-ABF2(RT) |  | F: GCAGCAACCTGTGACTTTCA  R: GCCAAAGTGCCATTCATACC |
| MD05G1082000-ABF4(RT) |  | F: AATAATGTTGCTGCCGGTTC  R: GCCTTACAACTGGTGGCTGT |
| proMdRAV1-HIS2 | *Sma*Ⅰ, *Sac*Ⅰ | F: cccgggTGTTCTTGTTTCGTTGATT  R: GAGCTCCTTTGTATTTTCTTTATCCT |
| proMdABI3-HIS2 | *EcoR*Ⅰ, *Sac*Ⅰ | F: GAATTC CTTACATTAACCATTGGTAC  R: GAGCTCTGTCAGGACGACGCTCATCC |
| proMdABI4-HIS2 | *Sma*Ⅰ, *Sac*Ⅰ | F: cccggg AGGGTGAGGAGGCGGCGAGC  R: GAGCTC TTCCTGCACAACACATAAAG |
| proMdABI3-1300-GUS | *Sal*Ⅰ, *Sma*Ⅰ | F: gaattcCTTACATTAACCATTGGTAC  R: cccggg TGTCAGGACGACGCTCATCC |
| proMdABI4-1300-GUS | *Sal*Ⅰ, *Sma*Ⅰ | F: gaattc AGGGTGAGGAGGCGGCGAGC  R: cccggg TTCCTGCACAACACATAAAG |
| MD16G1047700-MdRAV1-probe |  | F: TCTTTTTATATAAGTGGTCAAATTTTTTAT  R: AGAAAAATATATTCACCAGT TTAAAAAATA |
| MD13G1046100-MdRAV2-probe |  | F: CAATCCTCTGGGTTGACCCACCACAACCAC  R: GTGGTTGTGGTGGGTCAACCCAGAGGATTG |
| MD10G1169900-MdABI3-probe |  | F: AATTATTCAAGAACAACAAAATAAGTAAAA  R: TTTTACTTATTTTGTTGTTCTTGAATAATT |
| MD07G1224400-MdABI4-probe |  | F: TATAATTGTACCAACAACTTATTTATAGT  R: ACTATAAATAAGTTGTTGGTACAATTATA |
