# Supplementary material for: The MdWRKY31 transcription factor binds to the MdRAV1 promoter to mediate ABA sensitivity
Source: Hortic Res. 2019 Jun 1;6:66. doi: 10.1038/s41438-019-0147-1 (PMC6544635; doi:10.1038/s41438-019-0147-1)
Supplement: Supplementary file 7 — Supplement table 3. MdRAV1-1 (MD13G1046100) and MdRAV1-2 (MD16G1047700) promoter cis-acting element analysis [file 41438_2019_147_MOESM7_ESM.doc]

**Supplement table 3**. *MdRAV1-1* (MD13G1046100) and *MdRAV1-2* (MD16G1047700) promoter cis-acting element analysis.

*MdRAV1-1* (MD13G1046100) promoter cis-acting element analysis

| *Cis*-element name | Sequence | Function | Start site/bp | Termination site/bp |
| --- | --- | --- | --- | --- |
| ABRE | ACGTG | Cis-acting element involved in the abscisic acid responsiveness | 1715 | 1720 |
| LTR | CCGAAA | Cis-acting element involved in low-temperature responsiveness | 1499 | 1505 |
| G-box | TACGTG | Cis-acting regulatory element involved in light responsiveness | 1715 | 1721 |
| MBS | CAACTG | MYB binding site involved in drought-inducibility | 643 | 649 |
| W box | TTGACC | WRKY binding site | 1648 | 1654 |

*MdRAV1-2* (MD16G1047700) promoter cis-acting element analysis

| *Cis*-element name | Sequence | Function | Start site/bp | Termination site/bp |
| --- | --- | --- | --- | --- |
| ABRE | ACGTG | Cis-acting element involved in the abscisic acid responsiveness | 1589 | 1594 |
| AuxRR-core | TACCTGG | Cis-acting regulatory element involved in auxin responsiveness | 1602 | 1609 |
| [CGTCA-motif](http://bioinformatics.psb.ugent.be/webtools/plantcare/cgi-bin/show_site_info.htpl?QWhere=ID_of_Site like 'CGTCA'&StartAt=0&NbRecs=10) | CGTCA | Cis-acting regulatory element involved in the MeJA-responsiveness | 883 | 888 |
| G-box | TACGTG | Cis-acting regulatory element involved in light responsiveness | 1588 | 1594 |
| MBS | CAACTG | MYB binding site involved in drought-inducibility | 865 | 871 |
| W box | TTGACC | WRKY binding site | 1706 | 1712 |
